# Distinct BOLD Activation Profiles Following Central and Peripheral Oxytocin Administration in Awake Rats

**DOI:** 10.3389/fnbeh.2015.00245

**Published:** 2015-09-17

**Authors:** Craig F. Ferris, Jason R. Yee, William M. Kenkel, Kelly Marie Dumais, Kelsey Moore, Alexa H. Veenema, Praveen Kulkarni, Allison M. Perkybile, C. Sue Carter

**Affiliations:** ^1^Center for Translational NeuroImaging, Northeastern University, Boston, MA, USA; ^2^Kinsey Institute, Indiana University, Bloomington, IN, USA; ^3^Neurobiology of Social Behavior Laboratory, Department of Psychology, Boston College, Chestnut Hill, MA, USA

**Keywords:** oxytocin, cerebellum and emotion, olfaction and emotion, nucleus of the solitary tract, functional magnetic resonance imaging

## Abstract

A growing body of literature has suggested that intranasal oxytocin (OT) or other systemic routes of administration can alter prosocial behavior, presumably by directly activating OT sensitive neural circuits in the brain. Yet there is no clear evidence that OT given peripherally can cross the blood–brain barrier at levels sufficient to engage the OT receptor. To address this issue we examined changes in blood oxygen level-dependent (BOLD) signal intensity in response to peripheral OT injections (0.1, 0.5, or 2.5 mg/kg) during functional magnetic resonance imaging (fMRI) in awake rats imaged at 7.0 T. These data were compared to OT (1 μg/5 μl) given directly to the brain via the lateral cerebroventricle. Using a 3D annotated MRI atlas of the rat brain segmented into 171 brain areas and computational analysis, we reconstructed the distributed integrated neural circuits identified with BOLD fMRI following central and peripheral OT. Both routes of administration caused significant changes in BOLD signal within the first 10 min of administration. As expected, central OT activated a majority of brain areas known to express a high density of OT receptors, e.g., lateral septum, subiculum, shell of the accumbens, bed nucleus of the stria terminalis. This profile of activation was not matched by peripheral OT. The change in BOLD signal to peripheral OT did not show any discernible dose–response. Interestingly, peripheral OT affected all subdivisions of the olfactory bulb, in addition to the cerebellum and several brainstem areas relevant to the autonomic nervous system, including the solitary tract nucleus. The results from this imaging study do not support a direct central action of peripheral OT on the brain. Instead, the patterns of brain activity suggest that peripheral OT may interact at the level of the olfactory bulb and through sensory afferents from the autonomic nervous system to influence brain activity.

## Introduction

Oxytocin (OT) and OT-like peptides are evolutionarily conserved molecules found in all vertebrates [for review, see Gimpl and Fahrenholz (2001)]. Made in neurons localized to the paraventricular, supraoptic nuclei and accessory nuclei of the hypothalamus, OT can be released into the systemic circulation through axons terminating in the neurohypophysis of the pituitary gland. As a classical neurohormone, OT functions in childbirth to enhance uterine contractions and in breast feeding to promote milk letdown. OT neurons in the hypothalamus also make efferent connections to multiple areas of the brain, e.g., olfactory bulbs, basal ganglia, thalamus, and amygdala. As a neurotransmitter, OT functions in a variety of species-specific social behaviors associated with sexual behavior, social recognition, pair bonding, and maternal care [see reviews Pedersen (1997), Insel and Young (2001), Carter and Keverne (2002), Numan and Insel (2003), and Veenema and Neumann (2008)]. Collectively, as a chemical signaling system, the OT released peripherally into the blood stream and that released centrally in the brain can act independently of one another or in concert (Landgraf and Neumann, 2004).

As a neurohormone, circulating in the blood stream, there are no compelling data to support the notion that physiological levels of OT can re-enter the brain to affect behavior. Nonetheless, several studies in rodents report that peripheral administration of OT reduces anxiety-like behavior (de Wied et al., 1987; Uvnas-Moberg et al., 1994; McCarthy et al., 1996; Ring et al., 2006; Klenerova et al., 2009; Ayers et al., 2011; Bowen et al., 2011), promotes analgesia (Kang and Park, 2000), alters circadian feeding patterns (Zhang and Cai, 2011), increases sexual receptivity in rodents (Arletti and Bertolini, 1985; Moody et al., 1994), and promotes prosocial behavior (Ramos et al., 2013; Suraev et al., 2014; Calcagnoli et al., 2015; Holley et al., 2015). Interestingly, OT given centrally, either as a site-specific microinjection or intracerebroventricularly (ICV), has similar anxiolytic (Slattery and Neumann, 2010; Windle et al., 1997; Bale et al., 2001; Ring et al., 2006), analgesic (Lundeberg et al., 1994), circadian (Zhang and Cai, 2011) behavioral (Arletti and Bertolini, 1985; Caldwell et al., 1986), sexual (Witt et al., 1992), and prosocial (Williams et al., 1992; Lukas et al., 2011) effects. However, the mechanisms through which peripherally administered OT can affect behavior remain to be specified.

This question takes on greater importance considering the use of OT in the clinic. The high-profile publication of Kosfeld and coworkers showing that intranasal OT could enhance trust (Kosfeld et al., 2005), raised the possibility that this peptide given peripherally could be used to treat autism and other psychiatric conditions involving social cognition, affiliation, and bonding. Indeed, intranasal OT can decrease anxiety and increase vagal tone between parents and infants (Feldman and Eidelman, 2007), increase social learning, experience of attachment security, and social and cognitive empathy in less socially adept men (Buchheim et al., 2009; Bartz et al., 2010; Hurlemann et al., 2010), reduce the positive and negative signs of schizophrenia while improving verbal learning (Feifel et al., 2010), improve social recognition, social behavior, and affect in patients with autism spectrum disorders (Andari et al., 2010; Guastella et al., 2010), reduce sensitivity to social threat (Bertsch et al., 2013; Puglia et al., 2015), attenuate the fear response (Puglia et al., 2015), and increase extinction to conditioned fear (Eckstein et al., 2014). However, similar to the rodent studies, there is no evidence to date that intranasal OT enters the brain in levels sufficient to directly engage OT receptors or affect behavior. Thus, the mechanism of behavioral and emotional action remains unknown.

The purpose of the present study was to compare the pattern of brain activation measured in awake rats by blood oxygen level dependent (BOLD) imaging after exposure to OT given ICV or by intraperitoneal (IP) injection. If peripheral OT is able to access OT-sensitive neural circuits in the brain to affect behavior, we predicted that ICV and IP OT would activate many of the same brain areas, especially those containing high levels of OT receptors. We also predicted that peripheral OT would be associated with a dose-dependent change in brain activity or behavior either by direct activation of central OT receptors or indirectly through a pharmacological ligand/receptor interaction on a peripheral OT signal transduction pathway.

## Materials and Methods

### Animals

Adult, male Sprague Dawley rats were purchased from Charles River Laboratories (Wilmington, MA, USA). Animals were housed in Plexiglas cages (two per cage) and maintained in ambient temperature (22–24°C) on a 12:12 light:dark cycle (lights on at 09:00 a.m.). Food and water were provided *ad libitum*. All animals were acquired and cared for in accordance with the guidelines published in the *NIH Guide for the Care and Use of Laboratory Animals*. All methods and procedures described below were pre-approved by the Northeastern University Institutional Animal Care and Use Committee.

### Oxytocin preparation and administration

Oxytocin was purchased from Sigma Chemical (St Louis, MO, USA) and dissolved in 0.9% NaCl for IP injections or artificial cerebrospinal fluid for ICV injections. For the IP injections, rats were randomly assigned to one of four groups corresponding to saline vehicle, 0.1, 0.5, or 2.5 mg/kg OT. To deliver drug remotely during the imaging session, a poly-ethylene tube (PE-20), approximately 30 cm in length, was positioned in the peritoneal cavity. The range of doses of OT taken from the literature (Kang and Park, 2000; Ring et al., 2006) results in peripheral levels of peptides believed to be high enough to pass the blood–brain barrier and engage the brain OT receptor.

Intracerebroventricular injections were administered through a guide cannula placed in a small aperture on the skull. While under isoflurane anesthesia, rats were implanted with a 26-gage cannula of poly-ethylene tubing placed into the lateral cerebroventricle (2.5 mm lateral to the bregma and 5 mm below the dura). OT (1 μg) was given in a volume of 5 μl of artificial CSF during the scanning session. The rationale and methods for ICV injections in rats during imaging have been described in detail previously (Febo et al., 2004) and the dose of OT chosen was shown to increase BOLD signal in the OT neural circuit in lactating rats (Febo et al., 2005).

### Acclimation for awake imaging

To reduce the stress associated with head restraint, rats were acclimated to the restraining system (head holder and body tube) 1 week prior to their actual imaging session. The design of the restraining system included a padded head support obviating the need for ear bars helping to reduce animal discomfort while minimizing motion artifact. These acclimation sessions were run each day for four to five consecutive days. Rats were briefly anesthetized with 2–3% isoflurane while being secured into the head holder. The forepaws were secured with surgical tape. When fully conscious, the imaging system was placed into a black opaque box “mock scanner” for 30 min with a tape-recording of the MRI pulse sequence to simulate the bore of the magnet and the imaging protocol. A significant decline in respiration, heart rate, motor movements, and plasma corticosterone has been measured when the first and last acclimation periods are compared (King et al., 2005). The reduction in autonomic and somatic measures of arousal and stress improves the signal resolution and quality of the images.

### Image acquisition

**Figure 1:**
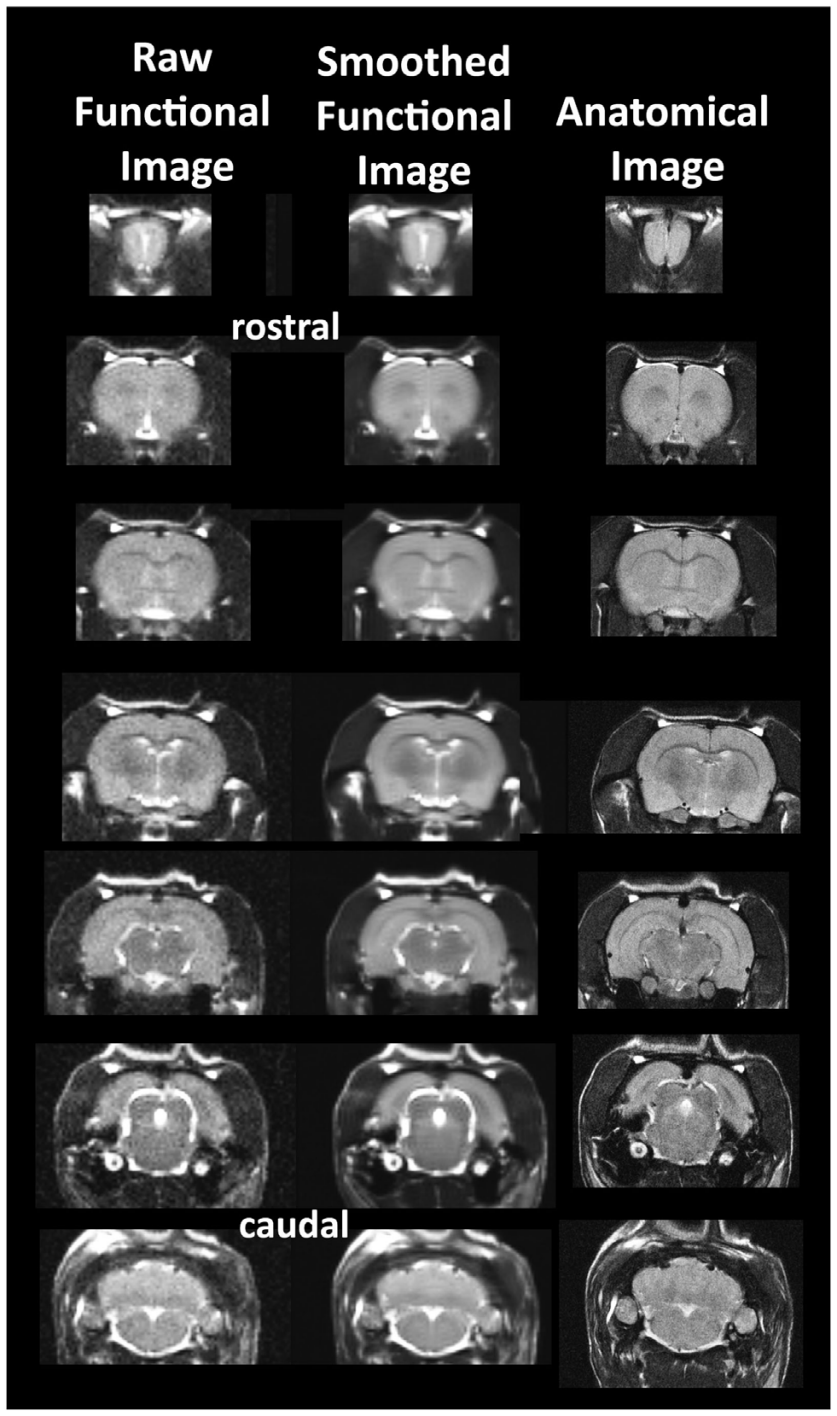
**Neuroanatomical fidelity**. Shown are representative examples of brain images collected during a single imaging session using a multi-slice spin echo, RARE (rapid acquisition with relaxation enhancement) pulse sequence. The column on the right shows axial sections collected during the anatomical scan taken at the beginning of each imaging session using a data matrix of 256 × 256, 22 slices in a field of view of 3.0 cm. The column on the left shows the same images but collected for functional analysis using HASTE, a RARE pulse sequence modified for faster acquisition time. These images were acquired using the same field of view and slice anatomy but a larger data matrix of 96 × 96. The images in the middle column have been smoothed during pre-processing. Note the anatomical fidelity between the functional images and their original anatomical image. The absence of any distortion is necessary when registering the data to atlas to resolve 171 segmented brain areas.

Animals were scanned at 300 MHz using a quadrature transmit/receive volume coil built into the rat head holder and restraining system for awake animal imaging (Animal Imaging Research, Holden, MA, USA). A video of the rat preparation for imaging is available at www.youtube.com/watch?v=JQX1wgOV3K4. The design of the coil provided complete coverage of the brain from olfactory bulbs to brain stem with excellent B1 field homogeneity. Experiments were conducted using a Bruker Biospec 7.0 T/20-cm USR horizontal magnet (Bruker, Billerica, MA, USA) and a 20-G/cm magnetic field gradient insert (ID = 12 cm) capable of a 120-μs rise time. At the beginning of each imaging session, a high-resolution anatomical data set was collected using the RARE pulse sequence [22 slice; 1.0 mm; field of view (FOV) 3.0 cm; matrix size 256 × 256; repetition time (TR) 2.5 s; echo time (TE) 12 ms; NEX 2; 3 min acquisition time]. Functional images were acquired using a multi-slice half-Fourier acquisition single-shot turbo spin echo (HASTE) pulse sequence. Bruker *Paravision* automatically finds the basic frequency, shims, power requirements for 90° and 180° pulses and sets the receiver gain. A single scanning session acquired 22 slices, 1.0 mm thick, every 6.0 s (TR), using an effective TE of 48 ms, FOV 3.0 cm, matrix size 96 × 96, NEX 1, and repeated 250 times for a total scanning time of 25 min. The in-plane pixel resolution was 312 μm^2^. Each scanning session was continuous, starting with 50 baseline image acquisitions, followed by vehicle or OT administration and continuing for another 200 image acquisitions. It should be noted that the ICV experiments did not acquire images from the olfactory bulb as was the case of the IP injections. This difference was due to a slice acquisition of 20 for ICV and 22 for IP. It should also be emphasized that high neuroanatomical fidelity and spatial resolution are critical in identifying distributed neural circuits in any animal imaging study. Many brain areas in a segmented rat atlas have in-plane boundaries of less than 400 μm^2^ and may extend for over 1000 μm in the rostral/caudal plane. With the development of a segmented, annotated 3D MRI atlas for rats (Ekam Solutions, Boston, MA, USA), it is now possible to localize functional imaging data to precise 3D “volumes of interest” in clearly delineate brain areas. Therefore, it is critical that the functional images are a very accurate reconstruction of the original brain neuroanatomy as shown in Figure 1.

The HASTE sequence, a spin-echo multislice pulse sequence used in these studies, corrects for field inhomogeneity, susceptibility artifact, chemical shift, and other imaging distortions and does not require any additional shimming as would be the case for gradient-echo pulse sequences which are commonly used in BOLD imaging studies. The major disadvantage to the HASTE sequence as compared to gradient echo is loss of signal contrast. The problem of sensitivity can be addressed with higher field strengths as used here (7 T) where the BOLD signal becomes a function of dynamic dephasing from diffusion of water at the level of the capillaries (Duong et al., 2003; Norris, 2006). Using multislice fast spin-echo sequences, the signal contrast with BOLD imaging is a function of T2 and not T2* at high field strengths. The extravascular signal surrounding capillary beds and small vessels is more reflective of the metabolic changes in brain parenchyma than signal from large draining veins helping to improve the localization of the signal changes (Yacoub et al., 2007). The BOLD signal is linear and reproducible at stimulus intervals of 1 s (Zhang et al., 2009). Positive BOLD signal represents an increase in oxygenated blood content when compared to baseline, and negative BOLD a decrease. Sustained negative BOLD may be attributed to a decrease in cerebral blood flow due to neural inhibition or redistribution of blood flow into neighboring regions of high activity (“vascular steal”) (Kim and Ogawa, 2012).

### Data analysis

Data were co-registered to a mean functional image using SPM8’s co-registrational code with the following parameters: quality, 0.97; smoothing, 0.35 mm; and separation, 0.5 mm. Gaussian smoothing was performed with a FWHM of 0.8 mm. Preprocessed functional files were then exported to Medical Image Visualization and Analysis (MIVA) for registration and segmentation. Images were aligned and registered to a 3D rat brain atlas, which is segmented and labeled with 171 discrete anatomical regions. Note the reduced slice acquisition in the ICV experiment eliminated the olfactory bulb and reduced the number of anatomical regions of the rat atlas to 163. The alignment process was facilitated by an interactive graphic user interface. The registration process involved translation, rotation, and scaling independently and in all three dimensions. Matrices that transformed each subject’s anatomy were used to embed each slice within the atlas. All pixel locations of anatomy that were transformed were tagged with regions of interest in the atlas. This combination created a fully segmented representation of each subject within the atlas. The inverse transformation matrix [Ti]-1 for each subject (i) was also calculated.

For voxel-based analysis, the percent change in BOLD signal for each independent voxel was averaged for all subjects with a baseline threshold of 2% BOLD change to account for normal fluctuation of BOLD signal in the rat brain under the awake condition (Brevard et al., 2003). A composite image of the whole brain representing the average of all subjects was constructed for each group for ROI analyses, allowing us to look at each ROI separately to determine the BOLD change and the number of activated voxels in each ROI. The *t*-test statistics used a 95% confidence level, two-tailed distributions, and heteroscedastic variance assumptions. As a result of the multiple *t*-test analyses performed, a false-positive detection controlling mechanism was introduced (Genovese et al., 2002). This subsequent filter guaranteed that, on average, the false-positive detection rate was below our cutoff of 0.05. Statistical *t*-tests were performed on each voxel (ca. 15,000 in number) of each subject within their original coordinate system. The average signal intensity in each voxel of the first 5 min of baseline (1–50) was compared to 5–15 min (acquisitions 51–150) and 15–25 min (acquisitions 151–250) post OT. Volume of activation was compared across experimental groups using the non-parametric Kruskal–Wallis test statistic. The brain areas were ranked in order of their significance. Brain areas were considered statistically different between experimental groups when comparison produced *P*-values less than or equal to our cutoff of 0.05. *Post hoc* analyses were performed with a Wilcoxon rank-sum test.

### Oxytocin receptor binding

Because of the pattern of BOLD activation seen in the olfactory bulb (see Results), OT receptor autoradiography was performed on the olfactory bulb of male Sprague Dawley rats. Rats were anesthetized under CO_2_ and decapitated. Brains were removed and quickly frozen in methylbutane on dry ice, and stored at −80°C. Olfactory bulbs were cut into 16-μm axial sections on a cryostat and mounted on slides. The receptor autoradiography procedure was performed according to Lukas et al. (2010) using [^125^I]-Ornithine Vasotocin Analog (d(CH_2_)_5_[Tyr(Me)^2^,Thr^4^,Orn^8^,[^125^I]Tyr^9^-NH_2_]-OVTA; Perkin Elmer, USA) as tracer for the OTR. Briefly, the slides were thawed and dried at room temperature. Slides were then fixed in 0.1% paraformaldehyde and washed two times in Tris buffer (pH 7.4). The slides were then exposed to tracer buffer (Tris + 10 mM MgCl_2_, 0.1% BSA, and tracer) for 60 min, and then washed four times in Tris + MgCl_2_. The slides were then dipped in distilled water, dried, and exposed to film (Kodak) for 7 days.

## Results

### Central versus peripheral oxytocin

**Figure 2:**
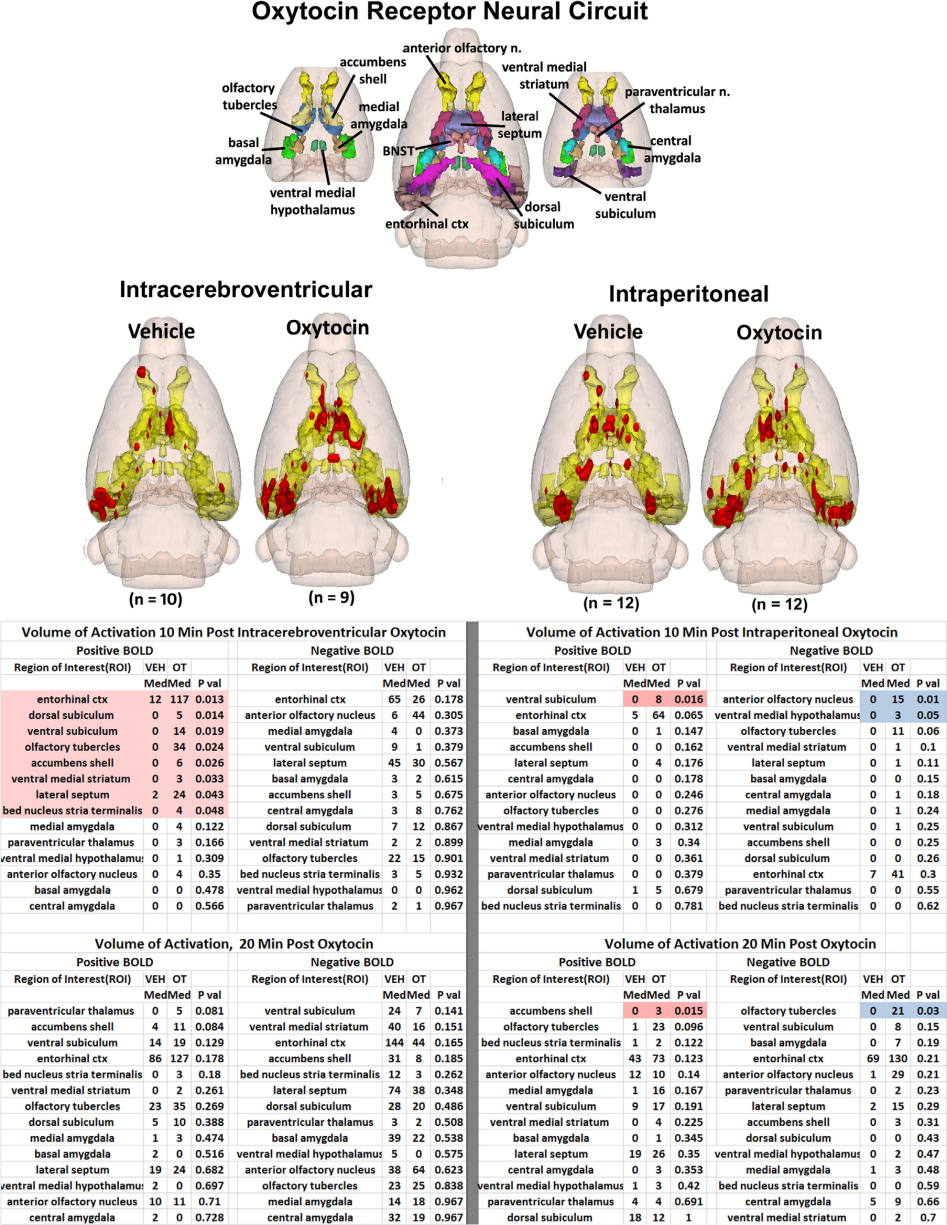
**Neural circuitry of high-density OT receptor binding sites**. The 3D color model at the top depicts the location of 14 brain areas in the rat reported to have a high density of OT receptor binding (De Kloet et al., 1985; van Leeuwen et al., 1985; Freund-Mercier et al., 1987; Tribollet et al., 1988). These areas have been coalesced into a single volume (yellow) as shown in the lower 3D images for ICV and IP administrations. Areas in red are the localization of the activated voxels comprising the composite average from the rats in each experimental group. Once fully registered and segmented, the statistical responses for each animal are averaged on a voxel-by-voxel bases. Those averaged voxels that are significantly different from baseline for positive BOLD are shown in their appropriate spatial location. Below are tables of these regions of interest for negative and positive BOLD at 10 and 20 min post ICV and IP OT. All areas are ranked in order of their significance. The brain areas highlighted in red and blue show significant changes in volumes of activation.

The 3D color model at the top of Figure 2 depicts the location of 14 brain areas in the rat reported to have a high density of OT receptor binding (De Kloet et al., 1985; van Leeuwen et al., 1985; Freund-Mercier et al., 1987; Tribollet et al., 1988; Dumais et al., 2013). These areas have been coalesced into a single volume (yellow) as shown in the lower 3D images for ICV and IP administrations of vehicle and OT. Areas in red are the composite average of the significant increase in volumes of activation (number of voxels in a ROI) for positive BOLD from all rats for each condition. The median (Med) number of positive and negative voxels activated for both vehicle and OT within 10 min of ICV and IP (2.5 mg/kg) injection are shown in the tables below. These brain areas are ranked in order of their significance. These data from all brain areas are presented in the 3D activation maps. Of the 14 areas having a high density of OT binding sites, eight were significantly activated by ICV OT as shown in the table below highlighted in red. Only one area, the ventral subiculum, was activated by IP OT within the first 10 min of injection. There was no significant change in negative BOLD for ICV OT; while IP OT reduced activity in the anterior olfactory nucleus and ventral medial hypothalamus shown highlighted in blue. By 20 min post ICV OT injection, there were neither positive nor negative changes in BOLD signal that reached significance. At the same time period, IP OT caused a significant increase in BOLD signal in the accumbens shell and decrease in signal in the olfactory tubercles.

The volume of activation for all brain areas ranked in order of their significance for ICV OT versus vehicle at 10 and 20 min post injection can be found in Tables S1 and S2 in Supplementary Material, respectively. Similarly, Tables S3 and S4 (Supplementary Material) show the volume of activation for all 171 brain areas for IP OT, 0.1 (*n* = 9), 0.5 (*n* = 13), and 2.5 (*n* = 12) mg/kg versus vehicle for 10 and 20 min post injection, respectively.

**Figure 3:**
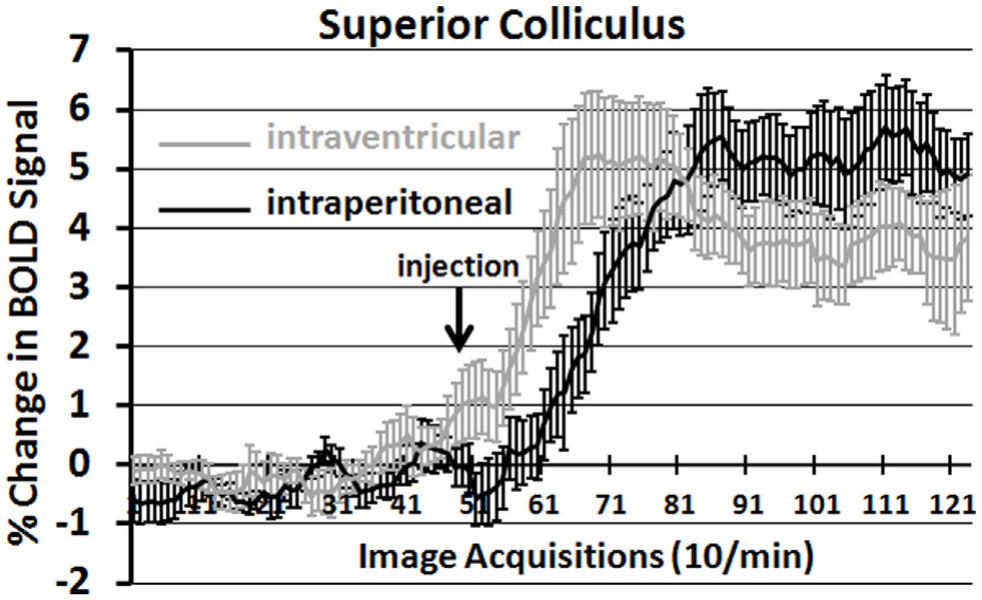
**Onset of BOLD signal change: intracerebroventricular versus intraperitoneal oxytocin**. Shown are the time-course plots for the change in BOLD signal in the superior colliculus following injection (arrow) of OT into the lateral cerebroventricle (gray, *n* = 8) or peritoneal cavity (black *n* = 11).

It should be noted that there is no clear dose-dependent change in the volume of activation for positive or negative BOLD at either time period for IP OT. A U-shaped pattern where OT doses of 0.1 and 2.5 are higher than the intermediate dose of 0.5 appears over most of the brain regions that show positive activation. Time-course plots showing the change in BOLD signal intensity in the superior colliculus for both ICV and IP administration of OT are presented in Figure 3. The superior colliculus was chosen because it showed robust activation with ICV (see Table S1 in Supplementary Material) and IP injections (see Table S3 in Supplementary Material). The change in BOLD signal occurs earlier with ICV injection by approximately 1 min as compared to IP (route × time: *F*_11,187_ = 37.64, *P* < 0.0001). The response peaks in around 2 min for ICV injection and about 4 min for IP injection.

### Activation of the primary olfactory system

**Figure 4:**
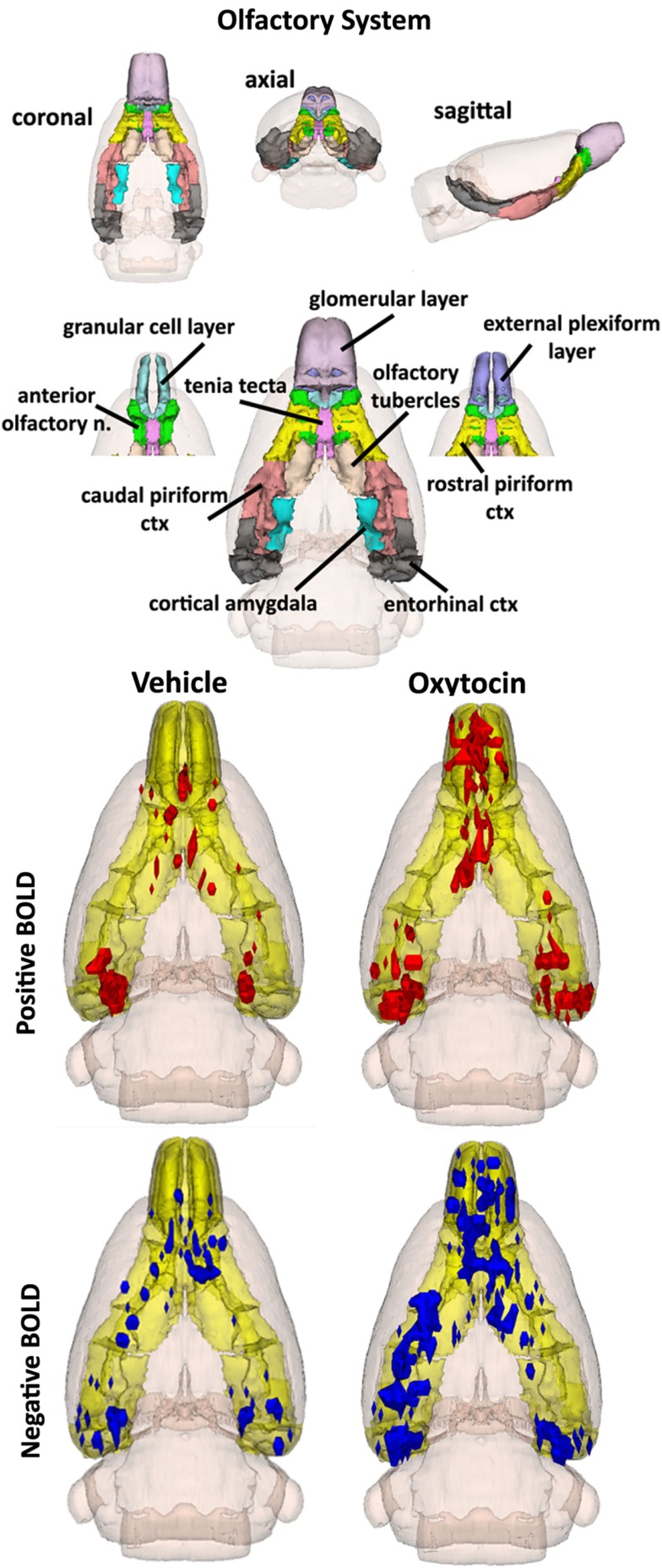
**The 3D color model at the top depicts the location of the brains that comprise the primary olfactory system in the rat**. These areas have been coalesced into a single volume (yellow) as shown in the lower 3D images for intraperitoneal OT (*n* = 12) and vehicle (*n* = 12). Areas in red (positive BOLD) and blue (negative BOLD) are the localization of the significantly changed voxels comprising the composite average from the rats in each experimental group.

By 20 min post IP OT administration, the number of activated brain areas is considerably reduced as compared to the first 10 min as shown in Table S3 in Supplementary Material. Several of the areas that remain significantly activated are part of the primary olfactory system (POS), e.g., external plexiform layer, glomerular layer, cortical amygdala, and rostral piriform cortex. Figure 4 shows 3D BOLD activation maps for vehicle and OT in the POS. The images at the top are color-coded and labeled for each 3D volume. As in Figure 2, these different volumes are coalesced into a single volume (yellow) showing the location in red of the average of the significant increase in volumes of activation (number of voxels in a ROI) for positive BOLD from all rats for each condition. These data are from the 2.5 mg/kg dose of OT acquired in the first 10 min post injection (see Tables S3 and S4 in Supplementary Material). Note the high activation of both positive and negative BOLD in the olfactory bulb (granular cell layer, glomerular layer, external plexiform layer) with OT treatment as compared to vehicle.

**Figure 5:**
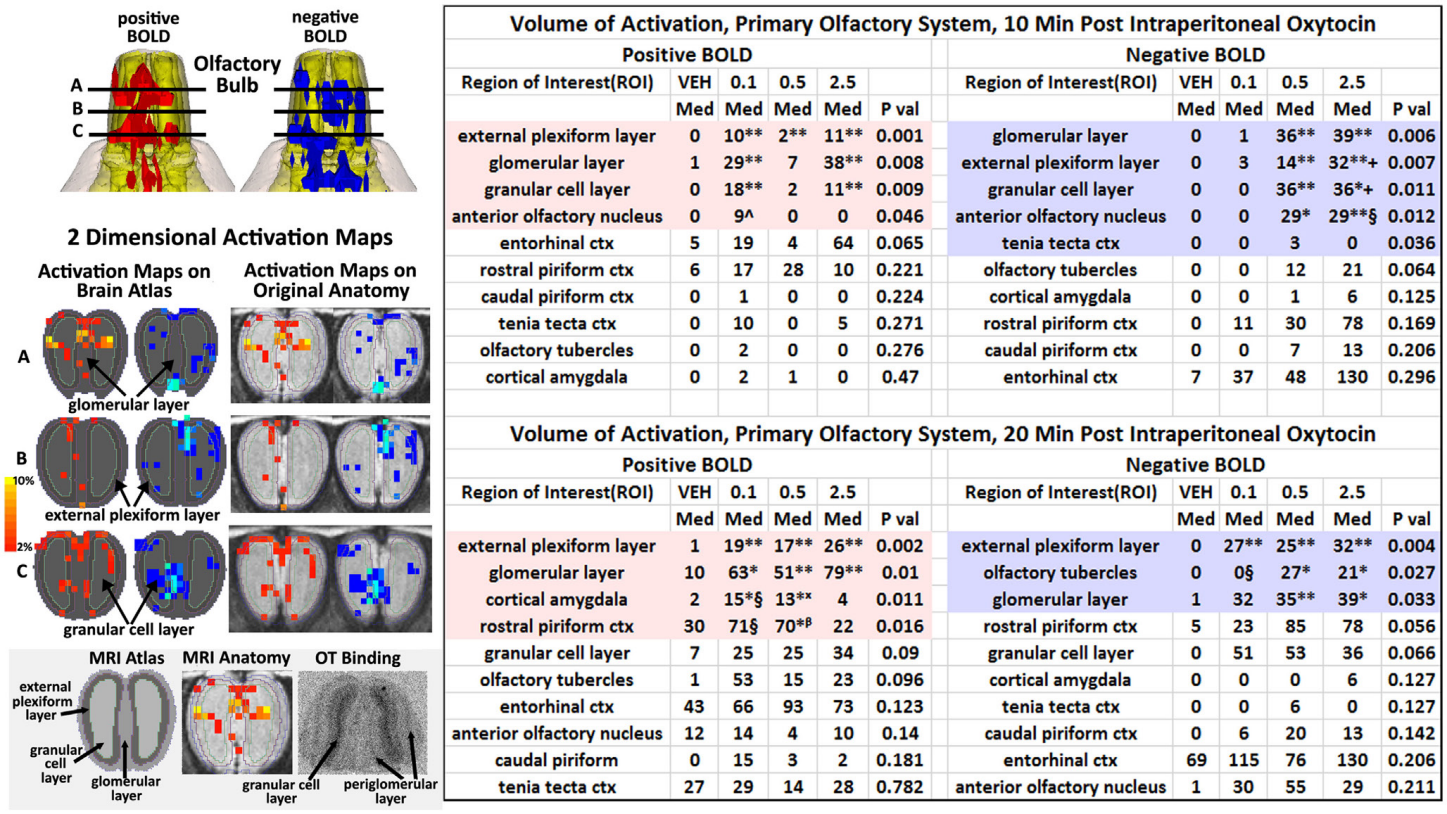
**Intraperitoneal oxytocin and the olfactory bulb**. The 3D images in the top left corner are magnifications of the olfactory bulbs shown in Figure 4. Below on the left are 2D activation maps from the rat brain atlas showing the precise location of the significantly altered positive (red) and negative (blue) voxels following IP OT (2.5 mg). The figures on the right show the localization of the voxels on the original neuroanatomical images. The vertical color strip indicates the percent change in BOLD signal. The lines (A, B, C) through the 3D top images show the approximate position of the axial 2D images below. The middle figure in the bottom panel shows the positive BOLD activation pattern on the original bulb neuroanatomy while the left figure shows the specific layers segmented and annotated as they appear in the rat atlas. The figure to right shows an autoradiogram of approximately the same section depicting OT binding in the granular layer (dark contrast). The tables show the median number of positive and negative for vehicle, 0.1, 0.5, and 2.5 IP OT at 10 and 20 min post IP injection. For the 0.1 mg dose positive BOLD, 10 and 20 min: *P < 0.05 **P < 0.01 compared to Veh; ^*P* < 0.05 compared to 2.5 mg; ^§^*P* < 0.05 compared to 2.5 mg. For the 0.5 mg dose positive BOLD 10 and 20 min: * < 0.05 **P < 0.01 compared to Veh; ^β^*P* < 0.05 compared to 2.5 mg. For the 2.5 mg dose positive BOLD, 10 and 20 min: **P < 0.01 compared to Veh. For the 0.1 mg dose negative BOLD, 20 min: **P < 0.01 compared to Veh; ^§^*P* < 0.05 compared to 2.5 mg. For the 0.5 mg dose negative BOLD 10 and 20 min: * < 0.05 **P < 0.01 compared to Veh. For the 2.5 mg dose negative BOLD, 10 and 20 min: *P < 0.05 **P < 0.01 compared to Veh; ^§^*P* < 0.05, +*P* < 0.01 compared to 0.1 mg.

**Figure 6:**
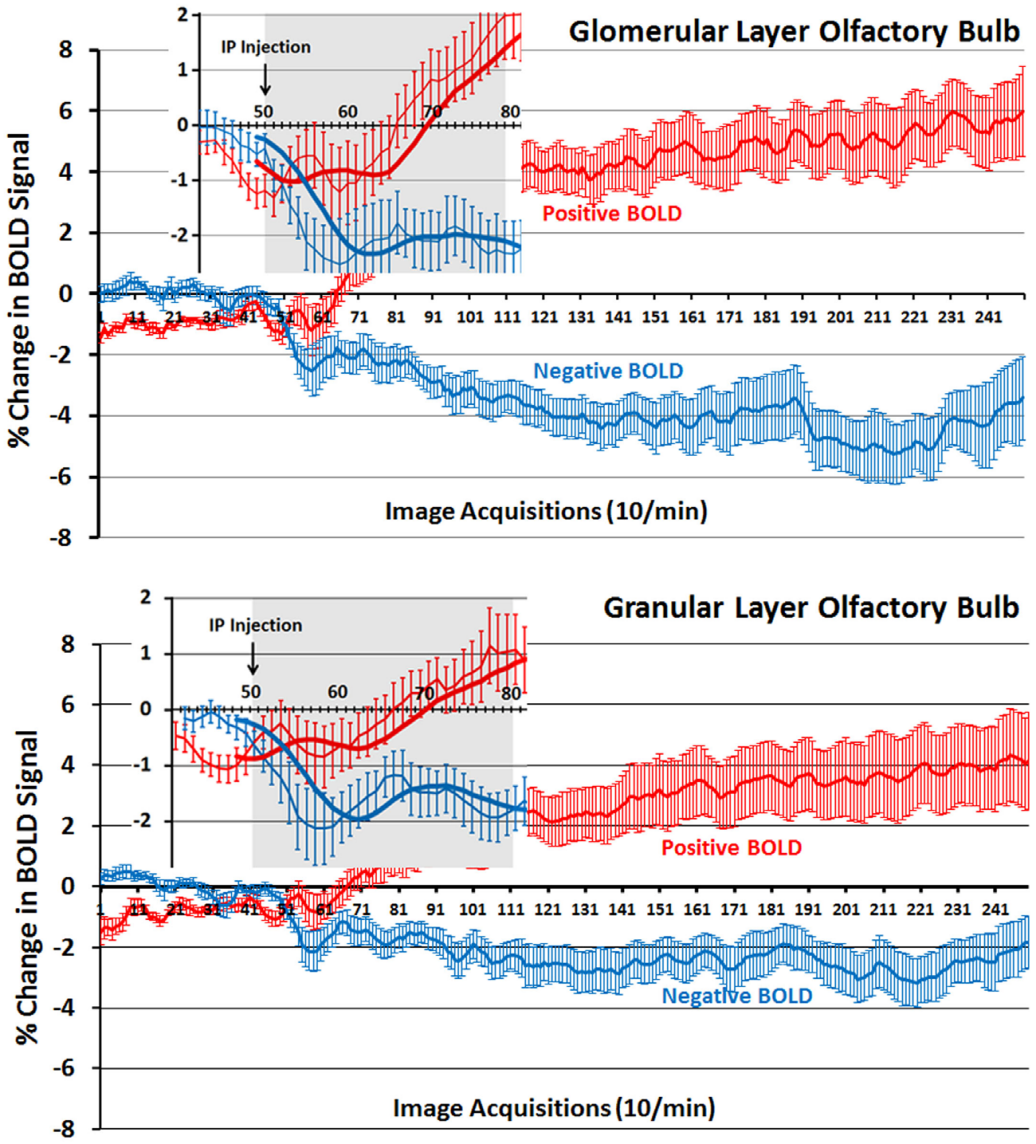
**Time-course plots for positive and negative BOLD in the olfactory bulb**. Shown are the changes in positive (red) and negative (blue) BOLD signals over time in the glomerular and granular layers of the olfactory bulb following intraperitoneal injection of OT. The insert is a magnified cut-out of the 3 min following injection. The thick lines show trend lines as an average of eight acquisitions. Vertical bars denote SEM.

The changes in positive and negative BOLD in the different areas of the olfactory bulb are better viewed in the 2D activation maps shown in Figure 5. At top is an enlargement of the olfactory bulbs depicting the 3D activation maps for positive and negative BOLD. The volumes of activation for each area of the bulb and the other areas of the POS for both the 10 and 20 min time periods are shown in the tables to the right. The data used to generate the 3D and 2D activation maps were taken from the vehicle and 2.5 mg/kg dose of OT collected during the 10-min time period. The precise location of the positive and negative voxels is shown registered to the MRI rat atlas and the original anatomy. These composites are the average number of voxels showing a significant increase above baseline for vehicle (*n* = 12) and 2.5 mg OT (*n* = 12). The approximate locations of the 2D axial sections relative to the 3D activation maps are indicated by the lines A, B, and C. It is clear from the position of positive and negative BOLD voxels that they do not overlap. Time-course plots showing the change in positive and negative BOLD signals in the glomerular and granular layers are presented in Figure 6. The insert shows a higher magnification of the first 3 min post IP injection of OT. Within the first minute, there is a significant decrease in negative BOLD over baseline for both glomerular layer (*t*_11_ = 4.27, *P* = 0.001) and granular layer (*t*_11_ = 4.14, *P* = 0.002). However, it is not until 3 min post injection that the positive BOLD significantly exceed baseline (glomerular: *t*_10_ = 3.70, *P* = 0.004; granular: *t*_10_ = 3.09, *P* = 0.01) showing the negative BOLD activation pattern precedes the positive BOLD in the olfactory bulb (negative versus positive × time: *F*_24,1056_ = 42.06, *P* < 0.0001). As noted above, Tables S3 and S4 in Supplementary Material show a high level of significant activity in many brain areas within the first 10 min of OT administration that is greatly reduced by 20 min post peptide. The major exception to this trend at 10 and 20 min post OT is the POS, particularly the olfactory bulbs. As shown in the tables attached to Figure 5, all doses of OT show significant changes in negative and positive BOLD from vehicle (*<0.05, **<0.01). Given the significant increase in BOLD activation in the olfactory bulb after IP OT, we performed receptor autoradiography to determine the presence of OT receptor binding in olfactory bulb layers. OT receptor binding was highest in the granular cell layer of the olfactory bulb. By contrast, there was faint OT receptor binding in the external plexiform layer and glomerular layer of the olfactory bulb. The distribution of OT receptor binding in the olfactory bulb is illustrated in Figure 5.

### Activation of brainstem and cerebellum

**Figure 7:**
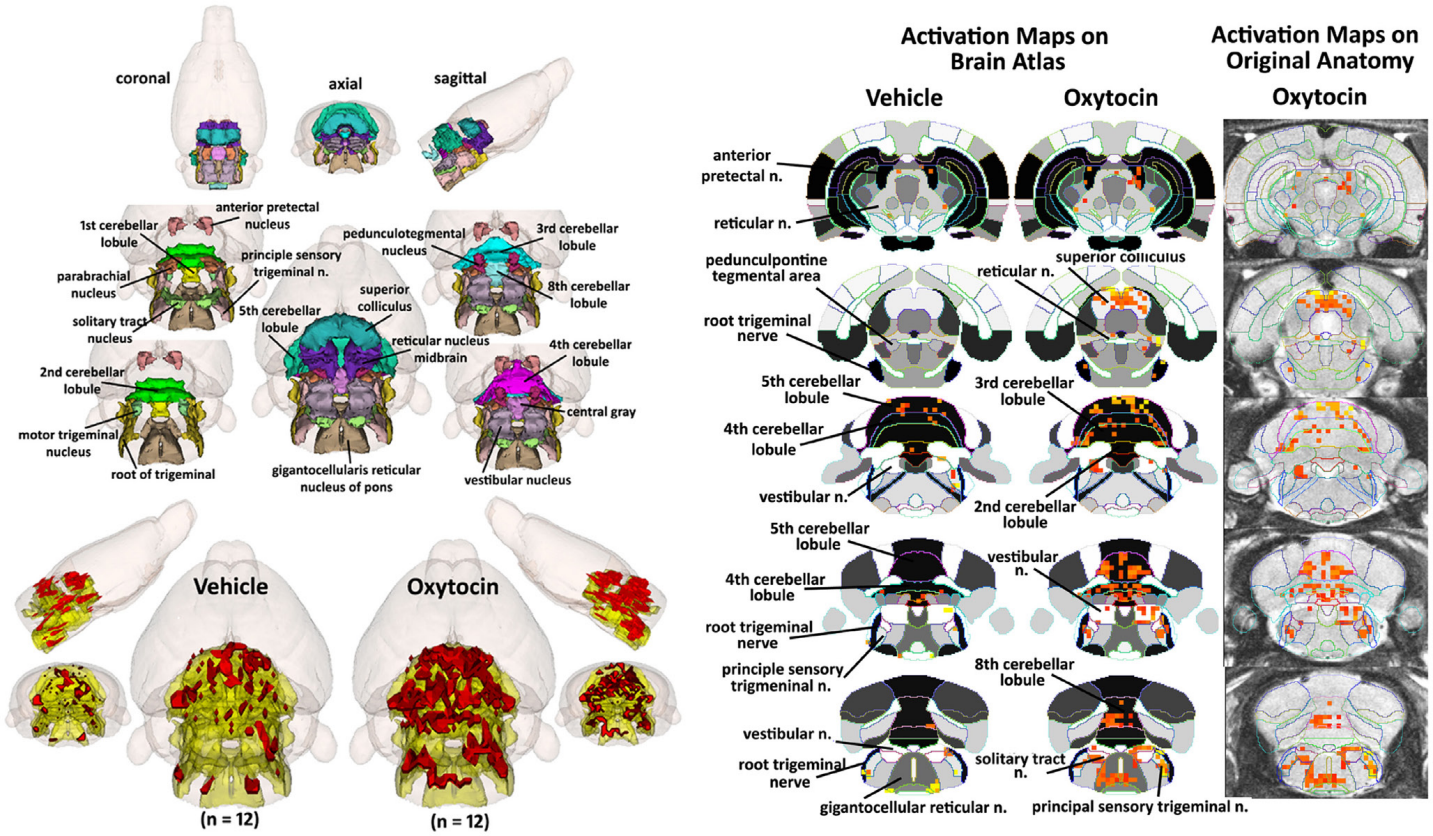
**Hindbrain areas activated by intraperitoneal oxytocin**. The 3D color model at the top left depicts the location of brain areas associated with activation of sensory afferents coming from the autonomic nervous system and synapses at the level of the brainstem and cerebellum. These areas have been coalesced into a single volume (yellow) as shown in the lower 3D images for IP vehicle (*n* = 12) and OT (*n* = 12). Areas in red are the localization of the activated voxels comprising the composite average from the rats in each experimental group following IP OT (2.5 mg). To the immediate right are 2D activation maps from the rat brain atlas showing the precise location of the significantly activated positive (red) voxels. The images to the far right show the localization of the voxels on the original neuroanatomical images.

How does one account for the many brain areas, particularly those localized to the brainstem, that are activated by IP OT? One hypothesis focuses on the notion that IP OT can stimulate OT receptors found on many peripheral organs, e.g., heart, blood vessels, urogenital system, etc. (Gimpl and Fahrenholz, 2001) that are innervated by the autonomic nervous system. Hence, Figure 7 shows a composite of several brain areas involved in processing viscero-sensory information from the autonomic nervous system. Many of these areas (gigantocellularis, nucleus of solitary tract, and parabrachial, pedunculotegmentum) have direct connections with viscero-sensory neurons in the spinal cord (Cechetto et al., 1985; Menetrey and Basbaum, 1987; Bernard and Besson, 1990; Menetrey and De Pommery, 1991; Krutki et al., 1999). The nucleus of the solitary tract (NST), trigeminal, and vestibular areas receive sensory information from the cranial nerves (Torvik, 1956; Caous et al., 2012). Included in Figure 7 are areas of the cerebellum that also receive viscero-sensory innervation from spinal cord and cranial nerves (Newman and Paul, 1969; Rubia, 1970; Nisimaru and Katayama, 1995; Krutki et al., 1999). As in Figures 2 and 4, the images are color-coded and labeled for each 3D volumes and coalesced into a single volume (yellow) showing the location in red of the significant increase in volumes of activation for positive BOLD from all rats for each condition. These data are from the 2.5 mg dose of OT acquired in the first 10 min post injection (see Table S3 in Supplementary Material). The precise location of the positive voxels is shown in the columns to the right registered to the 2D axial section from the MRI rat atlas and the original anatomy. These composites are the average number of voxels showing a significant increase above baseline for vehicle (*n* = 12) and 2.5 mg/kg OT (*n* = 12).

Table 1 shows these brainstem and cerebellar areas with viscero-sensory inputs ranked in order of their significance for positive and negative BOLD at time periods 10 and 20 min post IP OT administration. Note the absence of any significant negative BOLD for either time period. The significant difference in positive BOLD were limited only to the first 10 min period post OT. Also note that the significant increase in the volume of activation was primarily due to the 2.5 mg/kg dose of OT as compared to vehicle (*<0.05, **<0.01). The 0.1 mg/kg dose also showed a significant increase over vehicle for trigeminal areas, NST, reticular, and vestibular areas.

**Table 1:** **Activation in brainstem and cerebellum following intraperitoneal oxytocin**.

Positive BOLD						Negative BOLD					
Region of interest (ROI)	VEH	0.1	0.5	2.5		Region of interest (ROI)	VEH	0.1	0.5	2.5	

	Med	Med	Med	Med	*P*-value		Med	Med	Med	Med	*P*-value
**Volume activation 10 min post intraperitoneal oxytocin**											
Superior colliculus	8	23	0	91**^§^	0.003	Reticular area midbrain	0	0	3	9	0.117
Fourth cerebellar lobule	0	0	0	43**^β§^	0.003	Fifth cerebellar lobule	1	28	15	0	0.131
Anterior pretectal area	0	0	0	9**^β^^§^	0.004	Central gray	0	0	9	0	0.171
Fifth cerebellar lobule	3	4	0	113**^β^^§^	0.005	Fourth cerebellar lobule	0	4	0	0	0.208
Vestibular area	0	5*	0	22**^§^	0.006	Vestibular area	0	0	6	1	0.274
Reticular area midbrain	1	6^	0	10*^§^	0.01	Root of trigeminal nerve	0	0	12	9	0.29
Third cerebellar lobule	0	0	0	17^§^	0.011	Gigantocellular reticular pons	0	0	1	9	0.319
Root of trigeminal nerve	0	20**^	3	11*^x^	0.017	Eighth cerebellar lobule	0	0	0	0	0.365
Solitary tract nucleus	0	4*	0	5*^x^	0.023	Principal sensory trigeminal	0	0	2	5	0.378
Second cerebellar lobule	0	0	0	12*^x^	0.036	Parabrachial area	0	0	0	0	0.487
Principal sensory trigeminal	0	24*	4	12*	0.044	First cerebellar lobule	0	0	0	0	0.523
Parabrachial area	0	0	0	0	0.112	Superior colliculus	0	12	5	1	0.562
Pedunculopontine tegmentum	0	0	0	0	0.162	Third cerebellar lobule	0	2	4	1	0.586
Eighth cerebellar lobule	0	0	0	5	0.287	Second cerebellar lobule	0	1	1	0	0.654
Central gray	0	0	0	0	0.295	Solitary tract nucleus	0	0	0	0	0.692
First cerebellar lobule	0	0	0	0	0.554	Pedunculopontine tegmentum	0	0	0	0	0.817
Gigantocellular reticular pons	0	4	0	3	0.573	Anterior pretectal area	0	0	0	0	0.843
Motor trigeminal area	0	0	0	0	0.733	Motor trigeminal area	0	0	0	0	0.987
**Volume activation 20 min post intraperitoneal oxytocin**											
Reticular area midbrain	11	33	14	18	0.055	Third cerebellar lobule	7	18	0	6	0.08
Fourth cerebellar lobule	0	1	9	43	0.08	First cerebellar lobule	0	3	0	0	0.089
Solitary tract nucleus	0	5	2	9	0.081	Central gray	0	8	6	0	0.1
Eighth cerebellar lobule	0	0	0	13	0.094	Fourth cerebellar lobule	6	20	0	5	0.129
Fifth cerebellar lobule	16	17	32	73	0.114	Gigantocellular reticular pons	12	5	5	29	0.178
Motor trigeminal area	0	0	0	0	0.115	Reticular area midbrain	2	7	13	18	0.219
Parabrachial area	0	2	1	4	0.121	Fifth cerebellar lobule	42	65	3	24	0.293
Superior colliculus	50	69	59	82	0.134	Second cerebellar lobule	2	5	1	4	0.37
Root of trigeminal nerve	10	33	19	21	0.141	Root of trigeminal nerve	8	12	23	24	0.567
Vestibular area	3	13	16	22	0.175	Eighth cerebellar lobule	0	0	0	0	0.569
Principal sensory trigeminal	2	18	22	27	0.179	Motor trigeminal area	0	0	0	0	0.608
Anterior pretectal area	1	5	5	7	0.207	Vestibular area	4	17	3	2	0.626
Third cerebellar lobule	4	5	9	17	0.218	Principal sensory trigeminal	11	7	19	18	0.692
Pedunculopontine tegmentum	0	0	0	0	0.331	Parabrachial area	0	0	2	0	0.717
Second cerebellar lobule	8	13	8	26	0.338	Pedunculopontine tegmentum	0	0	0	0	0.744
Gigantocellular reticular pons	1	11	32	9	0.585	Solitary tract nucleus	0	0	0	2	0.775
Central gray	3	0	0	2	0.839	Superior colliculus	16	41	23	28	0.87
First cerebellar lobule	0	0	0	0	0.979	Anterior pretectal area	0	0	0	0	0.986

Shown are the median (Med) number of positive and negative voxels in brainstem and cerebellar areas affected 10 and 20 min following IP injections of vehicle (Veh *n* = 12), 0.1 (*n* = 9), 0.5 (*n* = 13), and 2.5 (*n* = 12) mg OT. The regions of interest are ranked in order of their significance. Probability values are presented on the far right column. The red highlights the significantly activated areas. The voxel numbers for each condition were analyzed using a Newman–Keuls multiple comparisons test statistic. For the 0.1 mg dose positive BOLD at 10: *P < 0.05 **P < 0.01 compared to Veh; ^*P* < 0.05 compared to 0.5 mg. For the 2.5 mg dose positive BOLD at 10 min: *P < 0.05 **P < 0.01 compared to Veh, ^§^*P* < 0.01 compared to 0.5 mg; ^β^*P* < 0.01 compared 0.1 mg.

## Discussion

### Central versus peripheral oxytocin

There are over 80 papers in humans studying the effects of ­intranasal OT on brain activity using functional MRI (http://www.ncbi.nlm.nih.gov/pubmed). Due to ethical considerations, there are no experiments in human subjects giving OT directly into the brain so it is not possible to compare imaging data from these two different routes of administration as we have done so here. In a previous study, we administered OT ICV to lactating female rats and found activation of many of the brain areas constituting the OT neural circuit involved in maternal behavior (Febo et al., 2005). Here, we provide the first data in animals looking at the effects of peripheral OT on brain activity using BOLD imaging. In these studies, we compared data from IP OT to those obtained from ICV OT. Both routes of administration used doses of OT reported to have robust effects on behavior. The doses for IP OT (0.1–2.5 mg/kg) were deliberately chosen from the high end of the spectrum to favor brain penetrance for behavioral effects (Newman and Paul, 1967; Kang and Park, 2000; Ring et al., 2006). If we assume that 0.002% (Mens et al., 1983) to 0.5% (Ermisch et al., 1985) of peripheral OT reaches the brain within 10 min after administration as reported, then our range of doses should be comparable to, or in excess of, our ICV dose of 1.0 μg.

In both routes of administration, there were significant changes in BOLD signal intensity within the first 10 min after OT injection. It has been argued in the animal literature that intranasal (Neumann et al., 2013) and IP (Ring et al., 2006) OT administration can directly affect OT neurotransmission in the brain. If so, we would expect that the ICV and IP injections of OT should show some similarity in brain activity. However, this was not the case. We used the density of OT receptors to identify the key nodes in a putative OT neural circuit as shown in Figure 2. The 14 brain areas shown in Figure 2 are not unique to rats but common to a variety of rodent species (Gimpl and Fahrenholz, 2001; Beery et al., 2008; Rosen et al., 2008; Campbell et al., 2009). While ICV OT significantly activated eight of these brain areas, IP OT activated only one. This would suggest that peripheral OT does not get into the brain in high enough concentration to effectively engage the OT receptor as compared to direct ICV OT within the first 20 min.

The question whether circulating OT from any peripheral route of administration can effectively cross the blood–brain barrier has been a topic of debate for many years (Churchland and Winkielman, 2012). While several studies have claimed elevations in CSF levels of OT following intranasal OT administration (Chang et al., 2012; Neumann et al., 2013; Striepens et al., 2013; Modi et al., 2014), it is not possible to distinguish OT released from endogenous stores, from OT given peripherally. There are several reports that intranasal OT is accompanied by an increase in plasma levels of OT (Landgraf, 1985; Cavdar et al., 2001; Gossen et al., 2012; Striepens et al., 2013). This coupled with the fact that OT given intravenously has many of the same behavioral effects reported for intranasal OT (Hollander et al., 2003, 2007; Bartz and Hollander, 2008) suggests that the intranasal and intravenous routes of administration in humans are acting on a peripheral transduction pathways to affect brain activity. This issue should be clearly resolved using quantitative chemical methods providing indisputable evidence that intranasal OT, or any other peripheral source of OT, achieves adequate levels in the brain to stimulate the OT receptor. These issues were thoroughly addressed in a study by Ludwig and colleagues comparing the neurobiological effects of ICV and intranasal vasopressin and OT in rats (Ludwig et al., 2013). The methodological concerns surrounding intranasal peptide administration have recently been reviewed by Leng and Ludwig (2015).

### Activation of the primary olfactory system

The most compelling finding in this imaging study was the significant activation of the POS by IP OT. There are numerous studies showing that the POS, indeed the olfactory bulb itself, is a critical brain area in mammals for processing and remembering socially relevant odors for use in social recognition and learning [review see Sanchez-Andrade and Kendrick (2009)]. Maternal behavior in rats and mice (Gandelman et al., 1971; Fleming and Rosenblatt, 1974), maternal recognition of offspring in sheep (Baldwin and Shillito, 1974), and social recognition in male rats (Dantzer et al., 1990) require the olfactory bulbs. The memory preservation of social recognition of juvenile female conspecifics is reduced with injection of OT receptor antagonist into the olfactory bulb (Larrazolo-Lopez et al., 2008) while OT infused in the olfactory bulbs of male rats preserves the memory of prior social interactions (Dluzen et al., 1998). Indeed, the olfactory bulb and its downstream connections to cortical amygdala, medial amygdala, BNST, and medial preoptic areas are all integrated in social recognition and all are sensitive to OT neurotransmission. It remains to be explored whether peripheral OT can gain direct access to the olfactory bulbs through the nasal epithelium as some predict (Guastella et al., 2013).

Oxytocin is not synthesized in neurons in the rat olfactory bulb (Tobin et al., 2010). However, there are OT fibers in the granular cell layer (Larrazolo-Lopez et al., 2008) that are thought to originate in the paraventricular nucleus of the hypothalamus (PVN) (Sawchenko and Swanson, 1982; Knobloch et al., 2012). Indeed, as shown in Figure 5, OT receptor binding is primarily confined to this area of the bulb. The granular cells are GABAergic and mingle with dendrites from mitral cells in the external plexiform layer. Activated granular cells inhibit mitral cell activity and output from the bulb to the anterior olfactory nucleus and olfactory cortex. It is thought that OT functions at the level of the bulb by inhibiting norepinephrine input coming into the granular layer (Sanchez-Andrade and Kendrick, 2009). The in-plane spatial resolution in these functional imaging studies was limited to 320 μm^2^ making it impossible to resolve the single layer of mitral cells that line the border between the external plexiform and glomerular layers; however, it is possible to see activity in the larger glomerular, external plexiform, and granular layers as shown in Figure 5. The first area of the olfactory bulb to receive input from the olfactory epithelium is the glomerulus. Based on the time-course data in Figure 6, the appearance of negative BOLD in both the glomerular and granular layers precedes the positive BOLD by close to 1 min. The granular cell layer shows a high level of significant negative BOLD during the first 10 min post OT and continues this trend over the 20 min imaging period (Figure 5). The glomerular layer, receiving direct inputs from sensory olfactory epithelium, remains significantly activated for both negative and positive BOLD over the 20 min imaging period as does the external plexiform layer. A significant activation of the cortical amygdala and rostral piriform cortex only appears at 20 min post IP OT. The sustained negative BOLD or diminished activity in the granular cell layer may account for the late activation of the olfactory cortex. Since norepinephrine excites granular cells which then inhibit mitral cells, OT could be decreasing noradrenergic transmission thereby increasing mitral cell activity and communication to efferent targets of the olfactory bulb.

### Activation of the nucleus of the solitary tract

As noted earlier, there are OT receptors in the male and female reproductive system, kidney, mammary glands, heart, vascular smooth muscle, and thymus (Gimpl and Fahrenholz, 2001). All of these organs are innervated by the autonomic nervous system. We hypothesized that following peripheral OT receptor activation, viscero-sensory information would be conveyed back to the brainstem through spinal cord and cranial nerves to affect BOLD signal intensity in many areas in the hindbrain associated with autonomic function. The NST, parabrachial and gigantocellularis nuclei, and reticular formation, key areas in the integration of viscero-sensory information, have direct sensory connections with spinal neurons (Menetrey and De Pommery, 1991). The anticipated areas of activation in the hindbrain from autonomic sensory fibers are shown in Figure 7. We did not see significant changes in BOLD signal in the parabrachial and gigantocellular areas with IP OT but several other areas, most importantly, the NST was activated. The NST is involved in the regulation of several visceral functions, particularly cardiovascular reflexes and vagal control of heart rate (Higa et al., 2002; Vela et al., 2010). Interestingly, central OT has an important role in regulating NST activity. The NST is high in both OT receptors and OT fibers (Buijs et al., 1990; Rinaman, 1998). OT neurons from the PVN have direct connections to the NST and function to facilitate vagal afferent transmission (Peters et al., 2008). Electrical stimulation of the PVN increases OT release in the NST (Landgraf and Neumann, 2004). Stimulation of vagal afferents increases activity in the PVN resulting in the release of OT in extrahypothalamic areas (Larrazolo-Lopez et al., 2008). The NST has extensive reciprocal connections with brain stem reticular activating system as well as the PVN (Ricardo and Koh, 1978; Herbert et al., 1990).

One intriguing hypothesis that finds support in this imaging study is OT activation of brain stem areas, particularly the NST, that would be involved in the “social engagement system” as proposed by Porges (2004). It is hypothesized that dysregulation of somatomotor function related to facial expression, visual tracking and gaze, head gestures, and auditory function can impact social and emotional behaviors. While higher cortical and subcortical areas control these somatomotor functions, afferent feedback from the vagus nerve to the NST affects the state of arousal and anxiety to impede or foster the activity of the social engagement system. This hypothesis has found support in several studies examining the physiologic state of infants with respect to the synchrony between mother–infant interactions (Moore and Calkins, 2004; Feldman, 2006; Feldman and Eidelman, 2007; Feldman et al., 2010). High vagal tone in infants, a measure of autonomic maturation and the ability to set a physiological state of “calmness,” assists in the establishing the mother–infant bond.

### Why the cerebellum?

We were surprised to see that many areas of the cerebellum were significantly activated with IP OT. Traditionally, the cerebellum was thought of as a brain structure involved in fine motor coordination; however, there are many early studies using stimulation and lesion techniques reporting that the cerebellum is involved in autonomic physiology, e.g., heart rate, blood pressure, respiration, piloerection (Snider and Maiti, 1976; Haines et al., 1984; Cavdar et al., 2001) and, more recently, a role in emotion and cognition (Newman and Paul, 1969; Haines et al., 1984; Perciavalle et al., 2013). There are reciprocal connections between the cerebellum and the hypothalamus, limbic cortex, amygdala, and hippocampus (Snider and Maiti, 1976; Heath et al., 1978; Haines et al., 1984; Calcagnoli et al., 2015). The cerebellum not only exercises efferent control of autonomic function but is also directly affected by visceral afferents via the splanchnic nerve (Newman and Paul, 1966, 1969; Langhof et al., 1973) and vagus nerve (Newman and Paul, 1966; Rubia and Phelps, 1970; Okahara and Nisimaru, 1991; Kondo et al., 1998). The cerebellum receives much of its innervation from the vestibular complex, the brain area relaying auditory information from the ear to the cortex. In addition to the cerebellum, the vestibular complex also has reciprocal connections with the NST (Jaarsma et al., 1997; Perciavalle et al., 2013) and the brainstem reticular formation which might explain its activation with peripheral OT. It was proposed by Cavdar and coworkers that the brainstem, particularly the NST, trigeminal, and vestibular nuclei together with the cerebellum interact with forebrain areas particularly the hypothalamus to regulate autonomic function (Cavdar et al., 2001). All of these brainstem areas were activated by IP OT. Hence, the cerebellum may be coordinating the autonomic response to the interoceptive environment activated by IP OT.

### Limitations and considerations in data interpretation

The stress associated with head restraint, restricted movement in the body tube, noise from the gradient coil, and the duration of the imaging session are all concerns when imaging awake animals. To address these problems, protocols have been developed for acclimating animals to the environment of the MR scanner and imaging procedure leading to a reduction in stress hormones levels and measures of autonomic activity regulated by the sympathetic nervous system [see review Ferris et al. (2011)]. Acclimation to the scanning session is achieved by putting subjects through several simulated imaging studies as described in the Section “Materials and Methods.” While it can be shown that signs of autonomic arousal and stress are reduced with acclimation, they are not eliminated entirely and that some stress is still likely with the imaging procedure. Thus, the OT-induced changes in BOLD signal intensity observed in these studies by either route of administration may have occurred against a backdrop of heightened arousal and stress over the 20 min imaging session.

There was no clear dose-dependent change in positive BOLD signal intensity to IP OT. The three doses of OT (0.1, 0.5, and 2.5 mg/kg) presented as a U-shaped curve with 0.5 mg/kg having little effect. Ramos and colleagues measuring social behavior in rats used similar doses of OT (0.1, 0.25, 0.5, and 1.0 mg/kg) given IP and failed to show a clear dose-response as only the 0.5 mg/kg dose had any significant effect (Ramos et al., 2013). The low dose of 0.1 mg/kg and high dose of 2.5 mg/kg used in our study brackets much of the behavioral literature although there are reports of behavioral effects with as little as 0.1–5.0 μg/kg of OT (Moody et al., 1994; Missig et al., 2010; Helena et al., 2011), a 1000-fold less than reported in most studies. OT has a very high binding affinity for its receptor (<0.5 nM) and the range of doses reported in the literature following systemic administration would easily engage the receptors – both central and peripheral, if the target could be reached. The broad range of peripheral doses used in behavioral studies, many of them pharmacologic as here, raises the possibility that OT could be interacting with other receptors. OT does have a moderate affinity for the arginine vasopressin V1a receptor (ca 100 nM) (Manning et al., 2012) and could be stimulating this signaling pathway to affect behavior. Evidence to this point comes from Ramos and colleagues showing that the behavioral effects they observed with OT treatment were also caused by vasopressin and that the effects of both peptides on social behavior could be blocked by a V1a receptor antagonist (Ramos et al., 2013). Similar results were reported for the hamster (Song et al., 2014). Hence, clear dose-dependent changes in behavior and in this case, BOLD signal, may be confounded by OT interacting with V1a receptors.

### Speculation

Interestingly, most of the activation, both positive and negative BOLD, occurred within the first 10 min post injection for both routes of OT administration. This raises a very interesting question. Why and how does a peptide with such a short half-life in the circulation (ca 1–5 min) (Meyer et al., 1987) have behavioral effects that last for hours as shown in myriad studies? While speculative, the answer may be found in the olfactory bulb, the only area in our study that remained activated over the 20 min imaging period (see Figure 5). Exogenous OT may set into motion central processes that sustain continued activation of POS. Thus, a burst of circulating OT may prime the olfactory bulb, which when combined with centrifugal inputs from the brain and autonomic nervous system via brainstem and cerebellum sustains a behavioral response in the context of relevant olfactory cues.

We can speculate further on the role of the cerebellum in OT-mediated behavior. Release of endogenous OT coordinates a visceral response to the relevant environmental stimulus which feeds back to the cerebellum creating an unconscious visceral template that reflects the intensity and valance of the stimulus. The stimulus is screened and evaluated at the level of the olfactory bulb – a function involving an interaction between OT and noradrenergic inputs from the locus coeruleus (Sanchez-Andrade and Kendrick, 2009). The coordinated emotional, cognitive, and behavioral response to the stimulus involves reciprocal connections between the hypothalamus, limbic system, hippocampus, brainstem, and cerebellum. The olfactory system and cerebellum share the top of this neural hierarchy. The cerebellum functions as the key area of the brain involved in integration of somatosensory information. Indeed, all pieces of somatosensory information have well-defined efferent connections to the cerebellum with the exception of the olfactory bulb. However, it remains a mystery why the cerebellum is consistently activated in human imaging studies that use an olfactory stimulus (Mainland et al., 2005). Moreover, the unknown pathway crosses over, e.g., lesions in the left cerebellum impair odor processing in the contralateral nostril (Mainland et al., 2005). Data based on changing odor intensity suggest that the intranasal trigeminal system may be responsible for odor-induced activation of the cerebellum (Iannilli et al., 2011).

Thus, we speculate that peripherally administered OT has behavioral effects by targeting OT receptors in the olfactory bulb and viscera. Exogenous OT need not cross the blood–brain barrier to engage central OT receptors and trigger OT neurotransmission. It needs to go only as far as the olfactory bulbs and visceral afferents. A pulse of OT, originating from the hypothalamic neurohypophyseal system that might naturally occur with emotional and biological events, readies the POS and coordinates an unconscious visceral response. The viscero-sensory information is integrated at the level of brain stem and stored in the cerebellum creating an internal model of the emotional/biological experience. The POS is constantly monitoring, evaluating, and screening information from the outside world associated with the emotional and/or biological event. The influence of the olfactory system on the organization and expression of behavior in animals is paramount and its effect on human behavior should not be under estimated.

## Conflict of Interest Statement

Craig F. Ferris has a financial interest in Animal Imaging Research, the company that developed the rat imaging system. Craig F. Ferris and Praveen Kulkarni have a financial interest in Ekam Solutions, the company that developed the mouse MRI atlas. The other authors declare no conflicts of interest.

## Supplementary Material

The Supplementary Material for this article can be found online at http://journal.frontiersin.org/article/10.3389/fnbeh.2015.00245
