# Supplementary material for: Distinct BOLD Activation Profiles Following Central and Peripheral Oxytocin Administration in Awake Rats
Source: Front Behav Neurosci. 2015 Sep 17;9:245. doi: 10.3389/fnbeh.2015.00245 (PMC4585275; doi:10.3389/fnbeh.2015.00245)
Supplement: Supplementary file 1 [file Table_1.PDF]

Table 1S Volume of Activation 10 Min Post Intracerebroventricular Oxytocin

Positive BOLD

Region of Interest(ROI)	CSF 5µl			OT 1µg/5µl			
	Med	Max	Min	Med	Max	Min	P val
lemniscal n.	0	15	0	21	35	4	0.001
5th cerebellar lobule	4	64	0	42	187	3	0.009
CA1 dorsal hippocampus	0	45	0	11	75	0	0.011
entorhinal ctx	12	138	0	117	303	0	0.013
pontine reticular n. oral	0	7	0	6	42	0	0.014
subiculum dorsal	0	27	0	5	24	0	0.014
inferior colliculus	1	56	0	51	119	0	0.017
anterior pretectal n.	0	13	0	5	24	0	0.017
ventral subiculum	0	19	0	14	47	0	0.019
6th cerebellar lobule	0	62	0	21	128	0	0.02
anterior cingulate area	12	18	0	34	118	0	0.021
medial septum	0	0	0	0	7	0	0.022
olfactory tubercles	0	23	0	34	71	0	0.024
simple lobule cerebellum	0	57	0	33	141	2	0.025
medial preoptic area	0	1	0	1	33	0	0.026
accumbens shell	0	6	0	6	21	0	0.026
diagonal band of Broca	0	4	0	4	15	0	0.029
medial dorsal thalamic n.	0	1	0	1	5	0	0.029
tenia tecta ctx	0	37	0	30	64	0	0.029
secondary motor ctx	5	87	0	68	123	0	0.032
ventral medial striatum	0	4	0	3	23	0	0.033
primary somatosensory ctx UL	0	72	0	2	23	0	0.034
dorsal lateral striatum	0	12	0	12	74	0	0.034
flocculus cerebellum	0	4	0	1	13	0	0.036
dentate gyrus ventral	0	24	0	4	25	0	0.037
prelimbic ctx	0.5	11	0	19	44	0	0.04
superior colliculus	0	61	0	30	97	0	0.04
anterior hypothalamic area	0	8	0	6	18	0	0.042
lateral septal n.	1.5	41	0	24	99	0	0.043
4th cerebellar lobule	0.5	10	0	13	96	0	0.044
dorsal medial striatum	0	24	0	5	38	0	0.046
CA3 dorsal hippocampus	0	18	0	21	55	0	0.047
bed n. stria terminalis	0	3	0	4	27	0	0.048
pontine nuclei	7.5	66	0	47	76	16	0.049
infralimbic ctx	0	33	0	18	58	0	0.052
reuniens n.	0	0	0	0	16	0	0.054
globus pallidus	0	0	0	0	20	0	0.054
paraventricular hypothalamic n.	0	0	0	0	8	0	0.054
ventral anterior thalamic n.	0	0	0	0	13	0	0.054
ventrolateral thalamic n.	0	0	0	0	7	0	0.054
interpeduncular n.	0	9	0	10	24	0	0.057
habenula n.	0	9	0	9	23	0	0.069
anterior thalamic nuclei	0	17	0	8	39	0	0.07
inferior olivary complex	0	22	0	11	30	0	0.072
CA2 hippocampus	0	1	0	0	3	0	0.073
anterior lobe pituitary	16	61	0	38	70	4	0.079
medial pretectal area	0	1	0	0	3	0	0.081
primary motor ctx	18	98	0	52	208	0	0.084
CA1 ventral hippocampus	0	18	0	2	55	0	0.084
3rd cerebellar lobule	0	25	0	15	104	0	0.094

Negative BOLD

Region of Interest(ROI)	CSF 5µl			OT 1µg/5µl			
	Med	Max	Min	Med	Max	Min	P val
10th cerebellar lobule	0	10	0	23	46	0	0.007
9th cerebellar lobule	0	8	0	10	65	0	0.01
copula of the pyramis	2.5	29	0	27	60	0	0.029
crus 1 of ansiform lobule	14	56	0	26	96	1	0.033
parafascicular thalamic n.	0	1	0	2	19	0	0.047
infralimbic ctx	6.5	36	0	0	16	0	0.052
lateral dorsal thalamic n.	0	7	0	1	8	0	0.059
interposed n.	1	19	0	9	15	0	0.074
2nd cerebellar lobule	9	38	0	22	61	0	0.078
medial pretectal area	0	2	0	0	0	0	0.082
precuniform n.	0	7	0	0	0	0	0.083
3rd cerebellar lobule	14	28	0	26	65	0	0.086
medial septum	2.5	7	0	0	4	0	0.098
central gray	0.5	23	0	10	43	0	0.105
dorsal raphe	0	5	0	1	7	0	0.119
neural lobe pituitary	0	0	0	0	5	0	0.126
vestibular n.	2.5	29	0	14	90	0	0.127
solitary tract n.	0	1	0	0	15	0	0.165
paraventricular hypothalamic n.	0	1	0	0	5	0	0.17
entorhinal ctx	65	236	0	26	131	0	0.178
trapezoid body	2	10	0	0	3	0	0.18
frontal association ctx	5	49	0	18	47	0	0.185
claustrum	1	22	0	0	5	0	0.186
auditory ctx	41	177	0	21	132	0	0.19
flocculus cerebellum	9	36	0	22	40	3	0.19
CA2 hippocampus	0	3	0	1	5	0	0.217
visual 2 ctx	22	121	0	10	51	0	0.219
paraflocculus cerebellum	43	114	5	83	202	6	0.22
pineal gland	1	3	0	3	6	0	0.227
reticular n.	1.5	11	0	5	37	0	0.229
CA3 dorsal hippocampus	9	30	0	15	103	0	0.234
magnocellular preoptic n.	0	10	0	0	5	0	0.235
supramammillary n.	1.5	8	0	5	8	0	0.238
temporal ctx	11	26	0	2	24	0	0.246
rostral piriform ctx	52	209	0	118	240	0	0.252
ectorhinal ctx	4	26	0	1	6	0	0.268
reuniens n.	0	6	0	0	18	0	0.276
cochlear n.	6.5	27	0	12	46	0	0.284
periaqueductal gray thalamus	37	95	0	43	111	0	0.286
reticulotegmental n.	0	0	0	0	1	0	0.292
suprachiasmatic n.	0	0	0	0	1	0	0.292
parabrachial n.	0	14	0	1	8	0	0.295
extended amydala	0	8	0	0	2	0	0.303
anterior olfactory n.	5.5	112	0	44	81	0	0.305
medial geniculate	15	36	0	26	45	0	0.32
parietal ctx	11	61	0	0	35	0	0.321
dorsal paragigantocellularis n.	0	4	0	0	20	0	0.321
interpeduncular n.	0	12	0	0	10	0	0.322
anterior thalamic nuclei	0.5	7	0	2	42	0	0.328
tenia tecta ctx	5.5	21	0	1	17	0	0.334

reticular n. midbrain	0	24	0	8	20	0	0.098
lateral amygdaloid n.	0	8	0	1	3	0	0.101
posterior hypothalamic area	0	12	0	0	15	0	0.103
root of trigeminal nerve	1	29	0	11	61	0	0.115
8th cerebellar lobule	0	34	0	5	51	0	0.119
reticular n.	0	9	0	3	16	0	0.119
medial amygdaloid n.	0	15	0	4	20	0	0.122
lateral orbital ctx	0	20	0	5	57	0	0.122
periaqueductal gray thalamus	0	41	0	1	44	0	0.122
lateral posterior thalamic n.	0	38	0	4	36	0	0.122
primary somatosensory ctx jaw	0	58	0	8	41	0	0.122
suprachiasmatic n.	0	0	0	0	1	0	0.125
accumbens core	0	0	0	0	7	0	0.126
claustrum	0	0	0	0	11	0	0.126
dorsomedial tegmental area	0	0	0	0	3	0	0.126
lateral preoptic area	0	0	0	0	2	0	0.126
parafascicular thalamic n.	0	0	0	0	20	0	0.126
raphe linear	0	0	0	0	3	0	0.126
magnocellular preoptic n.	0	3	0	0	4	0	0.126
paraflocculus cerebellum	5.5	73	0	32	175	0	0.127
facial n.	0	30	0	4	29	0	0.131
2nd cerebellar lobule	0	37	0	24	88	0	0.142
7th cerebellar lobule	0	20	0	1	16	0	0.143
secondary somatosensory ctx	0	40	0	0	8	0	0.155
ventral pallidum	0	9	0	0	7	0	0.155
central amygdaloid n.	0	3	0	0	8	0	0.166
paraventricular thalamic n.	0	12	0	3	17	0	0.166
raphe obscurus n.	0	6	0	0	0	0	0.168
pontine reticular n. caudal	0	5	0	0	65	0	0.17
CA3 ventral hippocampus	0	33	0	7	20	0	0.18
primary somatosensory ctx forelimb	0	28	0	8	39	0	0.187
vestibular n.	0	16	0	0	116	0	0.198
gigantocellular reticular n. pons	0	67	0	10	193	0	0.2
sub coeruleus n.	0	4	0	0	13	0	0.208
caudal piriform ctx	0	6	0	4	6	0	0.217
crus 1 of ansiform lobule	8	121	0	20	263	1	0.234
dorsal paragigantocellularis n.	0	6	0	0	18	0	0.235
ventral orbital ctx	0	4	0	0	49	0	0.257
crus 2 of ansiform lobule	0	90	0	10	105	0	0.262
perirhinal ctx	3	36	0	5	25	0	0.262
retrosplenial rostral ctx	17	135	0	36	124	0	0.27
parabrachial n.	0	10	0	1	18	0	0.275
extended amydala	0	0	0	0	11	0	0.292
endopiriform n.	0	0	0	0	13	0	0.292
intercalated amygdaloid n.	0	0	0	0	1	0	0.292
lateral cerebellar n.	0	0	0	0	7	0	0.292
locus ceruleus	0	0	0	0	2	0	0.292
median raphe n.	0	0	0	0	8	0	0.292
prerubral field	0	0	0	0	1	0	0.292
reticulotegmental n.	0	0	0	0	1	0	0.292
ventromedial thalamic n.	0	0	0	0	4	0	0.292
ventral posteriolmedial thalamic n.	0	0	0	0	1	0	0.292
primary somatosensory ctx shoulder	0	5	0	0	5	0	0.297
ventral tegmental area	0	9	0	0	11	0	0.298
6th cerebellar lobule	11	54	0	0	75	0	0.342
simple lobule cerebellum	7.5	70	0	16	75	0	0.345
triangular septal n.	0	2	0	0	12	0	0.362
prelimbic ctx	2	76	0	0	13	0	0.364
anterior cingulate area	26	134	0	5	104	0	0.368
dorsal medial striatum	35	130	0	60	123	0	0.368
central medial thalamic n.	0	2	0	0	8	0	0.37
lateral posterior thalamic n.	0	21	0	6	34	0	0.373
medial amygdaloid n.	4	29	0	0	13	0	0.373
ventral subiculum	8.5	35	0	1	16	0	0.379
principal sensory n. trigeminal	6.5	41	0	19	58	0	0.386
medial cerebellar n. fastigial	1	11	0	4	12	0	0.388
dorsomedial tegmental area	0	7	0	0	13	0	0.395
intercalated amygdaloid n.	0	1	0	0	2	0	0.398
facial n.	8.5	20	0	16	38	0	0.431
1st cerebellar lobule	4	17	0	9	22	0	0.451
habenula n.	4	13	0	10	13	0	0.453
8th cerebellar lobule	0	7	0	0	29	0	0.458
lateral orbital ctx	7.5	75	0	31	44	0	0.458
CA1 dorsal hippocampus	28	80	0	25	45	0	0.46
medial orbital ctx	0	7	0	0	26	0	0.461
lateral cerebellar n.	0	8	0	2	7	0	0.462
pontine reticular n. caudal	1.5	50	0	7	57	0	0.471
ventral tegmental area	3	15	0	4	19	0	0.476
locus ceruleus	0	3	0	0	1	0	0.52
raphe magnus	0	13	0	0	2	0	0.52
sub coeruleus n.	0	13	0	2	18	0	0.536
CA3 ventral hippocampus	9.5	39	0	14	41	0	0.538
globus pallidus	0	9	0	0	2	0	0.539
substantia nigra compacta	1.5	7	0	0	6	0	0.551
lateral amygdaloid n.	1	10	0	5	33	0	0.552
7th cerebellar lobule	0	4	0	0	1	0	0.563
inferior olivary complex	0	4	0	0	2	0	0.563
paramedian lobule	4.5	50	0	25	77	0	0.564
5th cerebellar lobule	25	86	0	29	123	5	0.567
lateral septal n.	45	79	0	30	142	0	0.567
posterior thalamic n.	1.5	28	0	4	13	0	0.58
lateral geniculate	3.5	29	0	13	49	0	0.581
cortical amygdaloid n.	0	19	0	0	3	0	0.585
secondary motor ctx	37	106	0	33	98	0	0.592
basal amygdaloid n.	2.5	37	0	2	51	0	0.615
medial preoptic area	0	14	0	0	22	0	0.617
dentate gyrus dorsal	21	82	0	30	79	0	0.623
primary motor ctx	34	277	0	22	132	0	0.623
4th cerebellar lobule	11	51	0	6	78	2	0.623
subthalamic n.	0	2	0	0	1	0	0.633
ventral anterior thalamic n.	0	8	0	0	7	0	0.635
red n.	0	10	0	0	8	0	0.636
premammillary n.	0	5	0	0	5	0	0.636
ventrolateral thalamic n.	0	21	0	0	20	0	0.637
medial mammillary n.	3	15	0	3	23	0	0.643
visual 1 ctx	21	136	0	22	36	0	0.65
accumbens shell	2.5	45	0	5	9	0	0.675
dorsal lateral striatum	11	117	0	15	76	0	0.678

ventral medial n.	0	14	0	1	19	0	0.309
insular ctx	0	121	0	2	63	0	0.31
substantia nigra reticularis	0.5	41	0	12	47	0	0.315
parvicellular reticular n.	0.5	46	0	11	137	0	0.315
auditory ctx	0	36	0	2	26	0	0.317
neural lobe pituitary	0	10	0	4	6	0	0.327
raphe magnus	0	3	0	0	13	0	0.345
anterior olfactory n.	0	45	0	4	15	0	0.35
primary somatosensory ctx barrel field	0	79	0	6	36	0	0.363
dentate gyrus dorsal	0	27	0	4	15	0	0.371
lateral hypothalamus	1	72	0	17	62	0	0.373
trapezoid body	0	5	0	0	10	0	0.399
posterior thalamic n.	0	1	0	0	22	0	0.403
ventral lateral striatum	0	10	0	2	116	0	0.412
paramedian lobule	0	60	0	4	74	0	0.436
primary somatosensory ctx hindlimb	2.5	24	0	6	28	0	0.451
cochlear n.	0	4	0	0	14	0	0.461
basal amygdaloid n.	0	3	0	0	7	0	0.478
interposed n.	0	5	0	0	10	0	0.48
temporal ctx	0	10	0	0	19	0	0.526
central medial thalamic n.	0	4	0	0	3	0	0.562
supramammillary n.	0	5	0	0	3	0	0.563
zona incerta	0	8	0	0	7	0	0.563
arcuate n. hypothalamus	0	5	0	0	7	0	0.571
9th cerebellar lobule	0	28	0	0	24	0	0.571
central gray	0	4	0	0	14	0	0.598
medial geniculate	0	23	0	0	14	0	0.607
medial orbital ctx	4.5	16	0	8	26	0	0.611
solitary tract n.	0	17	0	0	37	0	0.621
copula of the pyramis	0	45	0	0	26	0	0.637
frontal association ctx	4	41	0	3	23	0	0.644
principal sensory n. trigeminal	0	32	0	12	84	0	0.659
parietal ctx	2	30	0	0	21	0	0.691
premammillary n.	0.5	7	0	1	3	0	0.697
motor trigeminal n.	0	3	0	0	13	0	0.7
triangular septal n.	0	6	0	0	8	0	0.712
retrosplenial caudal ctx	1.5	48	0	4	43	0	0.73
rostral piriform ctx	16	63	0	21	79	0	0.743
ectorhinal ctx	0	3	0	0	13	0	0.766
pineal gland	0	3	0	0	3	0	0.818
10th cerebellar lobule	0	20	0	0	29	0	0.82
periolivary n.	0	19	0	0	11	0	0.85
substantia nigra compacta	0	4	0	0	4	0	0.891
primary somatosensory ctx trunk	0	9	0	0	3	0	0.909
1st cerebellar lobule	0	11	0	0	8	0	0.909
medial mammillary n.	0	18	0	0	6	0	0.925
lateral dorsal thalamic n.	0	3	0	0	4	0	0.925
visual 2 ctx	10	38	0	0	58	0	0.928
ventral posteriolateral thalamic n.	0	1	0	0	1	0	0.939
visual 1 ctx	11	46	0	9	41	0	0.967
cortical amygdaloid n.	2	16	0	1	30	0	1
dorsal raphe	0	5	0	0	4	0	1
lateral geniculate	0	13	0	0	6	0	1
medial cerebellar n. fastigial	0	10	0	0	6	0	1

inferior colliculus	41	134	0	62	130	2	0.683
root of trigeminal nerve	21	67	0	27	118	0	0.683
motor trigeminal n.	0	7	0	0	16	0	0.692
primary somatosensory ctx barrel field	37	165	0	30	119	0	0.713
accumbens core	0	53	0	0	22	0	0.716
CA1 ventral hippocampus	2	15	0	1	46	0	0.734
pontine nuclei	4.5	35	0	3	35	0	0.734
posterior hypothalamic area	2	19	0	5	12	0	0.738
zona incerta	3.5	33	0	8	36	0	0.738
gigantocellular reticular n. pons	4.5	17	0	6	26	0	0.741
primary somatosensory ctx forelimb	12	87	0	18	57	0	0.742
insular ctx	53	279	0	150	312	0	0.744
ventral orbital ctx	0	61	0	2	8	0	0.757
central amygdaloid n.	3	35	0	8	30	0	0.762
perirhinal ctx	23	60	0	18	60	0	0.775
secondary somatosensory ctx	14	116	0	11	69	0	0.775
ventromedial thalamic n.	0	21	0	0	15	0	0.792
lateral preoptic area	1	10	0	0	7	0	0.795
superior colliculus	21	98	0	28	61	0	0.805
retrosplenial rostral ctx	67	245	0	148	191	0	0.806
arcuate n. hypothalamus	0	7	0	0	10	0	0.82
primary somatosensory ctx trunk	0	17	0	0	6	0	0.82
endopiriform n.	2	61	0	3	31	0	0.832
primary somatosensory ctx shoulder	1	12	0	3	20	0	0.834
ventral posteriolateral thalamic n.	4	28	0	2	8	0	0.835
retrosplenial caudal ctx	13	73	0	18	60	0	0.836
caudal piriform ctx	10	63	0	28	62	0	0.836
primary somatosensory ctx upper lip	36	108	0	26	105	0	0.838
ventral posteriolmedial thalamic n.	1.5	28	0	1	15	0	0.863
anterior lobe pituitary	0.5	12	0	1	31	0	0.863
subiculum dorsal	6.5	46	0	12	35	0	0.867
primary somatosensory ctx hindlimb	10	79	0	16	45	0	0.867
lemniscal n.	4.5	27	0	5	16	0	0.868
primary somatosensory ctx jaw	33	117	0	53	120	0	0.869
ventral lateral striatum	15	155	0	12	90	0	0.869
reticular n. midbrain	23	153	0	23	129	0	0.87
crus 2 of ansiform lobule	0	10	0	0	17	0	0.882
ventral medial striatum	2	47	0	2	34	0	0.899
medial dorsal thalamic n.	2.5	12	0	2	15	0	0.899
olfactory tubercles	22	78	0	15	60	0	0.901
anterior amygdaloid n.	0	2	0	0	2	0	0.909
pedunculopontine tegmental area	0	11	0	0	2	0	0.909
raphe linear	0	11	0	0	10	0	0.921
diagonal band of Broca	0	9	0	0	9	0	0.925
prerubral field	0	5	0	0	11	0	0.925
anterior hypothalamic area	0	16	0	0	19	0	0.925
bed n. stria terminalis	3	14	0	5	22	0	0.932
ventral pallidum	4	59	0	8	45	0	0.932
pontine reticular n. oral	0	32	0	0	10	0	0.961
ventral medial n.	0	25	0	0	19	0	0.962
paraventricular thalamic n.	1.5	8	0	1	21	0	0.967
dentate gyrus ventral	13	39	0	14	29	0	0.967
anterior pretectal n.	0	4	0	0	1	0	1
lateral hypothalamus	15	98	0	16	112	0	1

**anterior amygdaloid n.**	**0**	**0**	**0**	**0**	**0**	**0**	**NaN**	**median raphe n.**	**0**	**6**	**0**	**0**	**5**	**0**	**1**
**dorsal medial n.**	**0**	**0**	**0**	**0**	**0**	**0**	**NaN**	**parvicellular reticular n.**	**6**	**23**	**0**	**12**	**31**	**0**	**1**
**precuniform n.**	**0**	**0**	**0**	**0**	**0**	**0**	**NaN**	**periolivary n.**	**4.5**	**21**	**0**	**6**	**33**	**0**	**1**
**pedunculopontine tegmental area**	**0**	**0**	**0**	**0**	**0**	**0**	**NaN**	**substantia nigra reticularis**	**8**	**31**	**0**	**16**	**28**	**0**	**1**
**red n.**	**0**	**0**	**0**	**0**	**0**	**0**	**NaN**	**dorsal medial n.**	**0**	**0**	**0**	**0**	**0**	**0**	**NaN**
**subthalamic n.**	**0**	**0**	**0**	**0**	**0**	**0**	**NaN**	**raphe obscurus n.**	**0**	**0**	**0**	**0**	**0**	**0**	**NaN**

Table 1S | Brain Activation with Intracerebroventricular Oxytocin 10 Minutes Post Injection
Shown are 163 brain areas and their median (Med), maximum (Max) and minimum (Min) number of positive and negative voxels affected 10 min following ICV injections of artificial cerebrospinal fluid (CSF) (n = 12) and OT (1 ug). The regions of interest are ranked in order of their significance. Probability values are presented on the far right column. The red and blue highlight the significantly activated areas for positive and negative BOLD, respectively. The voxel numbers for each condition were analyzed using a Newman-Keuls multiple comparisons test statistic. The yellow highlights mark brain areas that comprise the oxytocin receptor system shown in Fig 2. The gray highlighted columns aid in comparing the median values for each condition.
