# Supplementary material for: Distinct BOLD Activation Profiles Following Central and Peripheral Oxytocin Administration in Awake Rats
Source: Front Behav Neurosci. 2015 Sep 17;9:245. doi: 10.3389/fnbeh.2015.00245 (PMC4585275; doi:10.3389/fnbeh.2015.00245)
Supplement: Supplementary file 2 [file Table_2.PDF]

Table 2S Volume of Activation 20 Min Post Intracerebroventricular Oxytocin

Positive BOLD

Region of Interest(ROI)	CSF 5µl			OT 1µg/5µl			
	Med	Max	Min	Med	Max	Min	P val
medial septum	0	0	0	2	5	0	0.008
diagonal band of Broca	0	13	0	8	21	0	0.014
primary motor ctx	14	105	0	82	202	13	0.022
ventral anterior thalamic n.	0	0	0	0	14	0	0.022
pontine reticular n. oral	1	12	0	10	44	0	0.026
infralimbic ctx	1	46	0	31	56	5	0.036
raphe obscurus n.	0	6	0	0	0	0	0.039
lemniscal n.	11	28	0	23	39	4	0.041
secondary motor ctx	11	74	0	57	132	3	0.05
globus pallidus	0	0	0	0	22	0	0.054
raphe magnus	0	5	0	2	14	0	0.062
primary somatosensory ctx forelimb	0	25	0	5	37	0	0.063
prelimbic ctx	5	39	0	17	48	0	0.064
ventrolateral thalamic n.	0	1	0	0	12	0	0.065
anterior cingulate area	8	93	0	35	137	4	0.066
central medial thalamic n.	0	1	0	1	5	0	0.071
paraventricular thalamic n.	0	9	0	5	17	0	0.081
accumbens shell	3.5	13	0	11	25	0	0.084
medial dorsal thalamic n.	0	1	0	1	12	0	0.086
anterior thalamic nuclei	1	14	0	13	46	0	0.086
dorsal lateral striatum	0.5	25	0	21	80	0	0.086
6th cerebellar lobule	4	91	0	42	122	0	0.089
anterior pretectal n.	1.5	15	0	8	24	0	0.094
CA1 dorsal hippocampus	1.5	66	0	13	125	0	0.099
accumbens core	0	2	0	0	6	0	0.102
tenia tecta ctx	8	53	0	35	64	4	0.102
medial preoptic area	0	12	0	2	24	0	0.106
CA1 ventral hippocampus	0	32	0	13	51	0	0.11
suprachiasmatic n.	0	0	0	0	1	0	0.125
extended amydala	0	0	0	0	15	0	0.126
median raphe n.	0	0	0	0	12	0	0.126
medial geniculate	0	32	0	0	8	0	0.126
ventral subiculum	14	27	0	19	50	0	0.129
posterior hypothalamic area	0	11	0	6	21	0	0.133
CA2 hippocampus	0	1	0	0	4	0	0.137
simple lobule cerebellum	5	107	0	36	138	0	0.138
arcuate n. hypothalamus	1.5	9	0	0	3	0	0.141
precuniform n.	0	2	0	0	0	0	0.168
ventral orbital ctx	2	7	0	6	54	0	0.173
crus 1 of ansiform lobule	15	132	0	37	247	0	0.176
entorhinal ctx	86	170	0	127	282	0	0.178
bed n. stria terminalis	0	9	0	3	23	0	0.18
interpeduncular n.	4.5	14	0	11	24	0	0.188
ventral lateral striatum	5.5	14	0	0	142	0	0.203
5th cerebellar lobule	22	121	0	54	182	0	0.204
medial mammillary n.	2.5	22	0	0	4	0	0.21
primary somatosensory ctx jaw	0	80	0	3	59	0	0.21
supramammillary n.	0.5	6	0	0	2	0	0.217
triangular septal n.	1.5	9	0	0	8	0	0.22
anterior hypothalamic area	0	25	0	2	30	0	0.22
paraventricular hypothalamic n.	0	6	0	0	10	0	0.229
4th cerebellar lobule	5.5	34	0	16	85	0	0.234
CA3 dorsal hippocampus	2	57	0	20	67	0	0.245
primary somatosensory ctx upper lip	0	79	0	3	9	0	0.251
lateral orbital ctx	0	54	0	3	59	0	0.261
ventral medial striatum	0	27	0	2	25	0	0.261
reticular n.	1.5	15	0	4	17	0	0.262
olfactory tubercles	23	63	0	35	97	0	0.269
dorsomedial tegmental area	0	0	0	0	5	0	0.292
reticulotegmental n.	0	0	0	0	4	0	0.292
ventromedial thalamic n.	0	0	0	0	3	0	0.292
8th cerebellar lobule	2	34	0	13	53	0	0.299
2nd cerebellar lobule	14	39	0	24	82	0	0.302

Negative BOLD

Region of Interest(ROI)	CSF 5µl			OT 1µg/5µl			
	Med	Max	Min	Med	Max	Min	P val
medial mammillary n.	1.5	16	0	11	23	3	0.01
ectorhinal ctx	12	31	0	2	10	0	0.011
supramammillary n.	1.5	8	0	4	8	3	0.016
claustrum	15	29	0	0	13	0	0.019
raphe magnus	4	15	0	0	3	0	0.023
prelimbic ctx	28	122	0	0	13	0	0.026
primary motor ctx	187	324	0	56	175	0	0.045
medial septum	7.5	10	0	0	5	0	0.046
neural lobe pituitary	0	1	0	0	5	0	0.065
secondary motor ctx	92	222	0	30	126	0	0.072
triangular septal n.	1	14	0	5	12	0	0.08
10th cerebellar lobule	0	32	0	23	46	0	0.092
globus pallidus	5.5	31	0	2	9	0	0.099
magnocellular preoptic n.	4	10	0	0	9	0	0.102
ventral lateral striatum	123	276	0	28	140	0	0.102
anterior cingulate area	120	215	0	16	142	1	0.102
primary somatosensory ctx forelimb	80	161	0	26	80	0	0.111
7th cerebellar lobule	4	27	0	0	11	0	0.119
copula of the pyramis	9	37	0	24	64	0	0.12
primary somatosensory ctx hindlimb	50	104	0	24	58	0	0.12
visual 2 ctx	104	194	5	56	146	2	0.121
infralimbic ctx	20	79	0	1	27	0	0.121
extended amydala	6.5	17	0	0	12	0	0.121
cochlear n.	14	36	0	34	49	0	0.13
premammillary n.	0	5	0	1	5	0	0.131
anterior pretectal n.	5.5	21	0	0	22	0	0.131
parietal ctx	42	80	0	20	63	0	0.141
ventral subiculum	24	65	1	7	54	0	0.141
ventral medial striatum	40	88	0	16	49	0	0.151
medial pretectal area	0	5	0	0	1	0	0.152
median raphe n.	3.5	18	0	1	4	0	0.153
entorhinal ctx	144	254	10	44	342	0	0.165
precuniform n.	0	11	0	0	0	0	0.168
primary somatosensory ctx trunk	7	29	0	5	17	0	0.184
accumbens shell	31	97	0	8	51	0	0.185
diagonal band of Broca	4.5	16	0	0	15	0	0.187
1st cerebellar lobule	6	17	0	14	23	0	0.198
medial preoptic area	6.5	48	0	0	32	0	0.202
secondary somatosensory ctx	87	155	0	58	100	0	0.205
posterior hypothalamic area	14	30	0	6	22	0	0.218
primary somatosensory ctx shoulder	10	24	0	7	14	0	0.218
medial geniculate	21	39	0	38	47	0	0.219
central gray	11	21	0	17	44	0	0.219
dorsal lateral striatum	102	232	0	61	111	0	0.22
periaqueductal gray thalamus	71	129	0	90	138	0	0.236
interposed n.	3	18	0	13	19	0	0.248
gigantocellular reticular n. pons	26	145	0	9	54	0	0.249
endopiriform n.	34	81	0	11	58	0	0.249
parvicellular reticular n.	35	116	0	18	69	0	0.25
primary somatosensory ctx jaw	121	248	0	89	149	0	0.252
auditory ctx	137	235	6	106	168	4	0.253
lateral preoptic area	4.5	31	0	1	10	0	0.257
bed n. stria terminalis	12	63	0	3	28	0	0.262
lateral amygdaloid n.	8.5	20	0	15	29	0	0.267
ventromedial thalamic n.	16	39	0	1	33	0	0.276
cortical amygdaloid n.	7	44	0	2	31	0	0.281
primary somatosensory ctx barrel field	171	302	0	102	201	0	0.287
2nd cerebellar lobule	24	116	3	46	93	0	0.288
subthalamic n.	0.5	2	0	0	2	0	0.293
anterior amygdaloid n.	0	4	0	0	2	0	0.296
ventral orbital ctx	29	67	0	5	33	0	0.303
retrosplenial caudal ctx	46	89	0	20	78	0	0.306
medial cerebellar n. fastigial	2.5	11	0	9	13	0	0.314

premammillary n.	2.5	7	0	1	4	0	0.307
intercalated amygdaloid n.	0	1	0	0	0	0	0.343
pedunculopontine tegmental area	0	3	0	0	0	0	0.343
inferior olivary complex	11	25	0	14	31	0	0.345
parvicellular reticular n.	28	54	0	34	135	0	0.345
central gray	0	5	0	0	9	0	0.347
periolivary n.	5	36	0	0	21	0	0.364
reuniens n.	0	1	0	0	19	0	0.364
gigantocellular reticular n. pons	36	93	0	65	196	0	0.368
parabrachial n.	0.5	12	0	4	20	0	0.373
dentate gyrus ventral	3	36	0	8	46	0	0.379
insular ctx	0.5	268	0	8	118	0	0.379
subiculum dorsal	4.5	36	0	10	23	0	0.388
retrosplenial rostral ctx	22	205	0	42	126	0	0.39
paraflocculus cerebellum	38	132	0	68	158	0	0.391
primary somatosensory ctx barrel field	0	91	0	10	27	0	0.401
claustrum	0	2	0	0	11	0	0.403
copula of the pyramis	7.5	50	0	4	32	0	0.409
superior colliculus	18	100	0	49	86	0	0.413
motor trigeminal n.	0	3	0	0	19	0	0.423
pineal gland	0	2	0	0	3	0	0.423
medial cerebellar n. fastigial	0	15	0	0	8	0	0.427
rostral piriform ctx	45	227	0	30	72	0	0.437
visual 2 ctx	4	46	0	13	60	0	0.445
primary somatosensory ctx hindlimb	0.5	22	0	6	14	0	0.45
posterior thalamic n.	0	2	0	0	31	0	0.461
inferior colliculus	32	123	0	55	103	0	0.461
caudal piriform ctx	0	89	0	2	16	0	0.467
medial amygdaloid n.	1	20	0	3	16	0	0.474
dentate gyrus dorsal	1	68	0	4	37	0	0.476
raphe linear	0	2	0	0	5	0	0.48
ventral tegmental area	1.5	14	0	0	10	0	0.48
lateral posterior thalamic n.	2	39	0	11	25	0	0.481
solitary tract n.	1.5	20	0	9	37	0	0.497
basal amygdaloid n.	1.5	21	0	0	10	0	0.516
dorsal raphe	0	6	0	0	2	0	0.52
7th cerebellar lobule	0	21	0	11	19	0	0.536
zona incerta	0	11	0	0	8	0	0.562
3rd cerebellar lobule	11	56	0	13	98	0	0.567
crus 2 of ansiform lobule	9.5	93	0	14	111	0	0.592
temporal ctx	0.5	6	0	0	21	0	0.594
pontine reticular n. caudal	0	14	0	1	83	0	0.596
ventral posteriolateral thalamic n.	0	6	0	0	3	0	0.598
magnocellular preoptic n.	0	4	0	0	3	0	0.603
CA3 ventral hippocampus	2.5	37	0	5	18	0	0.618
paramedian lobule	14	66	0	17	79	0	0.619
lateral geniculate	0	37	0	0	11	0	0.636
periaqueductal gray thalamus	7	60	0	2	38	0	0.648
habenula n.	8	27	0	11	26	0	0.648
ectorhinal ctx	0	5	0	0	14	0	0.649
frontal association ctx	9.5	66	0	7	51	0	0.65
interposed n.	0	14	0	0	10	0	0.673
primary somatosensory ctx shoulder	0	5	0	0	3	0	0.673
lateral septal n.	19	86	0	24	81	0	0.682
anterior lobe pituitary	37	59	0	44	73	0	0.683
ventral medial n.	2	20	0	0	23	0	0.697
lateral cerebellar n.	0	2	0	0	7	0	0.699
cochlear n.	0	10	0	0	17	0	0.705
anterior olfactory n.	10	67	0	11	17	0	0.71
visual 1 ctx	5.5	35	0	6	37	0	0.711
sub coeruleus n.	0	6	0	0	28	0	0.716
central amygdaloid n.	1.5	4	0	0	9	0	0.728
dorsal paragigantocellularis n.	0	6	0	0	20	0	0.731
flocculus cerebellum	2	11	0	1	16	0	0.737
neural lobe pituitary	2	12	0	4	8	0	0.741
reticular n. midbrain	5	44	0	8	25	0	0.774
10th cerebellar lobule	3	25	0	0	30	0	0.791
trapezoid body	1	10	0	0	15	0	0.791

anterior lobe pituitary	2.5	13	0	4	47	0	0.319
ventral posteriolateral thalamic n.	22	47	0	6	48	0	0.322
substantia nigra reticularis	12	52	0	20	57	0	0.324
accumbens core	39	83	0	15	58	0	0.34
pontine reticular n. oral	6	64	0	11	27	0	0.345
3rd cerebellar lobule	28	88	0	45	119	0	0.348
lateral septal n.	74	169	0	38	152	0	0.348
primary somatosensory ctx upper lip	119	191	0	78	152	0	0.348
central medial thalamic n.	1.5	9	0	0	13	0	0.348
CA1 dorsal hippocampus	98	187	0	40	121	0	0.367
prerubral field	4	15	0	0	12	0	0.372
crus 2 of ansiform lobule	7.5	65	0	0	53	0	0.373
8th cerebellar lobule	3	66	0	0	57	0	0.377
dorsal raphe	2.5	8	0	5	9	0	0.377
visual 1 ctx	88	187	0	68	143	0	0.391
9th cerebellar lobule	3	64	0	12	74	0	0.406
raphe linear	5.5	17	0	4	14	0	0.407
6th cerebellar lobule	34	156	0	11	134	0	0.41
insular ctx	232	519	0	216	409	0	0.413
anterior hypothalamic area	6	40	0	0	27	0	0.427
posterior thalamic n.	41	96	0	14	70	0	0.431
vestibular n.	26	102	0	63	107	0	0.438
pineal gland	2.5	4	0	0	4	0	0.458
ventral pallidum	38	82	0	9	64	0	0.458
zona incerta	33	56	0	15	55	0	0.46
locus ceruleus	0	2	0	1	2	0	0.473
frontal association ctx	8	61	0	24	51	0	0.484
subiculum dorsal	28	56	0	20	49	0	0.486
lateral orbital ctx	43	142	0	34	72	0	0.487
reticulotegmental n.	0	5	0	0	4	0	0.488
reuniens n.	6.5	27	0	6	27	0	0.501
paraventricular thalamic n.	3	18	0	2	23	0	0.508
flocculus cerebellum	21	35	6	27	41	9	0.513
ventral posteriolmedial thalamic n.	25	65	0	4	56	0	0.534
lemniscal n.	14	31	0	8	40	0	0.536
basal amygdaloid n.	39	105	0	22	81	0	0.538
CA3 ventral hippocampus	22	48	0	33	50	0	0.538
temporal ctx	18	59	2	24	30	0	0.539
trapezoid body	3	16	0	4	13	0	0.557
simple lobule cerebellum	57	163	7	38	213	0	0.568
ventral medial n.	5	16	0	0	23	0	0.575
arcuate n. hypothalamus	0	6	0	0	10	0	0.585
anterior thalamic nuclei	3.5	49	0	4	47	0	0.592
parabrachial n.	4.5	42	0	7	37	0	0.592
pontine reticular n. caudal	31	136	0	15	98	0	0.595
medial orbital ctx	2.5	19	0	0	15	0	0.596
lateral geniculate	20	46	0	16	49	0	0.621
ventral tegmental area	7.5	19	0	9	17	0	0.621
facial n.	5	33	0	17	29	0	0.622
anterior olfactory n.	38	115	0	64	112	0	0.623
dorsal paragigantocellularis n.	4.5	22	0	0	22	0	0.644
dorsal medial n.	0	2	0	0	2	0	0.652
CA1 ventral hippocampus	14	88	0	16	47	0	0.653
perirhinal ctx	51	97	1	55	79	0	0.653
paraflocculus cerebellum	76	217	27	102	259	30	0.683
lateral dorsal thalamic n.	1.5	9	0	3	9	0	0.705
pedunculopontine tegmental area	1	13	0	4	7	0	0.706
solitary tract n.	1.5	23	0	3	14	0	0.706
ventral anterior thalamic n.	9.5	24	0	8	21	0	0.706
lateral cerebellar n.	4.5	11	0	3	7	0	0.708
motor trigeminal n.	6	29	0	6	27	0	0.709
dorsal medial striatum	112	199	0	106	136	0	0.713
reticular n. midbrain	83	196	0	53	205	3	0.713
red n.	4.5	12	0	1	9	0	0.732
lateral posterior thalamic n.	11	57	0	11	70	0	0.738
dorsomedial tegmental area	3.5	29	0	6	21	0	0.742
lateral hypothalamus	69	150	0	64	156	0	0.743
paramedian lobule	32	64	0	25	93	0	0.744

ventral pallidum	1	12	0	1	12	0	0.798
cortical amygdaloid n.	6.5	26	0	6	27	0	0.804
pontine nuclei	41	87	0	51	99	19	0.806
medial pretectal area	0.5	3	0	0	2	0	0.823
medial orbital ctx	13	22	0	11	22	5	0.837
principal sensory n. trigeminal	15	41	0	19	83	0	0.838
root of trigeminal nerve	18	66	0	28	68	2	0.838
dorsal medial n.	0	1	0	0	3	0	0.878
endopiriform n.	0	2	0	0	17	0	0.878
prerubral field	0	1	0	0	2	0	0.878
parietal ctx	0	19	0	0	20	0	0.882
lateral preoptic area	0	6	0	0	3	0	0.891
lateral amygdaloid n.	0	9	0	0	7	0	0.891
auditory ctx	1	58	0	0	33	0	0.895
facial n.	3.5	30	0	1	33	0	0.897
vestibular n.	3.5	21	0	0	122	0	0.897
substantia nigra compacta	1	8	0	1	4	0	0.898
1st cerebellar lobule	0	18	0	0	9	0	0.921
primary somatosensory ctx trunk	0	9	0	0	4	0	0.921
perirhinal ctx	5.5	56	0	7	47	0	0.934
subthalamic n.	0	1	0	0	1	0	0.939
parafascicular thalamic n.	0	5	0	0	31	0	0.962
9th cerebellar lobule	1	33	0	0	35	0	0.965
retrosplenial caudal ctx	0.5	53	0	1	22	0	0.966
dorsal medial striatum	3.5	53	0	5	38	0	0.967
lateral hypothalamus	4.5	87	0	9	79	0	0.967
substantia nigra reticularis	11	60	0	10	44	0	0.967
locus ceruleus	0	2	0	0	1	0	1
lateral dorsal thalamic n.	0	3	0	0	9	0	1
secondary somatosensory ctx	0	50	0	0	26	0	1
ventral posteriolmedial thalamic n.	0	4	0	0	1	0	1
anterior amygdaloid n.	0	0	0	0	0	0	NaN
red n.	0	0	0	0	0	0	NaN

interpeduncular n.	0	25	0	2	10	0	0.757
4th cerebellar lobule	37	99	0	33	119	9	0.775
inferior olivary complex	0	5	0	0	4	0	0.792
periolivary n.	9	27	0	5	26	0	0.803
reticular n.	7	35	0	11	33	0	0.805
5th cerebellar lobule	85	228	0	47	248	28	0.806
CA2 hippocampus	1.5	6	0	2	5	0	0.835
parafascicular thalamic n.	3.5	51	0	9	40	0	0.836
root of trigeminal nerve	46	99	5	40	110	0	0.838
olfactory tubercles	23	85	1	25	81	0	0.838
crus 1 of ansiform lobule	49	298	15	46	248	7	0.838
caudal piriform ctx	68	130	0	35	112	0	0.838
paraventricular hypothalamic n.	0	9	0	0	9	0	0.864
ventrolateral thalamic n.	16	56	0	19	42	0	0.868
sub coeruleus n.	13	55	0	8	44	0	0.87
superior colliculus	52	186	0	57	184	0	0.87
tenia tecta ctx	6.5	40	0	3	35	0	0.899
substantia nigra compacta	2	10	0	2	14	0	0.901
medial dorsal thalamic n.	4.5	19	0	4	23	0	0.902
CA3 dorsal hippocampus	30	87	0	49	121	0	0.902
dentate gyrus ventral	23	65	0	14	59	0	0.935
inferior colliculus	98	183	0	82	211	20	0.935
rostral piriform ctx	163	354	35	128	345	19	0.935
retrosplenial rostral ctx	151	257	0	143	287	1	0.935
suprachiasmatic n.	0	1	0	0	1	0	0.939
intercalated amygdaloid n.	1	3	0	1	3	0	0.966
medial amygdaloid n.	14	36	0	18	34	0	0.967
central amygdaloid n.	32	46	0	19	50	0	0.967
habenula n.	9.5	24	0	11	36	0	0.967
principal sensory n. trigeminal	32	78	4	46	89	0	0.967
dentate gyrus dorsal	42	117	0	67	96	0	1
pontine nuclei	6	90	0	8	43	0	1
raphe obscurus n.	0	0	0	0	0	0	NaN

Table 2S | Brain Activation with Intracerebroventricular Oxytocin 20 Minutes Post Injection.
Shown are 163 brain areas and their median (Med), maximum (Max) and minimum (Min) number of positive and negative voxels affected 10 min following ICV injections of artificial cerebrospinal fluid (CSF) (n = 12) and OT (1 ug). The regions of interest are ranked in order of their significance. Probability values are presented on the far right column. The red and blue highlight the significantly activated areas for positive and negative BOLD, respectively. The voxel numbers for each condition were analyzed using a Newman-Keuls multiple comparisons test statistic. The yellow highlights mark brain areas that comprise the oxytocin receptor system shown in Fig 2. The gray highlighted columns aid in comparing the median values for each condition.
