# Supplementary material for: Distinct BOLD Activation Profiles Following Central and Peripheral Oxytocin Administration in Awake Rats
Source: Front Behav Neurosci. 2015 Sep 17;9:245. doi: 10.3389/fnbeh.2015.00245 (PMC4585275; doi:10.3389/fnbeh.2015.00245)
Supplement: Supplementary file 3 [file Table_3.PDF]

TABLE 3S Volume of Activation 10 Min Post Intraperitoneal Oxytocin

Positive BOLD

Region of Interest(ROI)	VEH Med	0.1 Med	0.5 Med	2.5 Med	P val
external plexiform layer	0	10	2	11	0.001
superior colliculus	8	23	0	91	0.003
4th cerebellar lobule	0	0	0	43	0.003
arcuate hypothalamus	0	2	0	2	0.003
anterior pretectal area	0	0	0	9	0.004
5th cerebellar lobule	3	4	0	113	0.005
simple lobule cerebellum	0	2	0	99	0.006
vestibular area	0	5	0	22	0.006
inferior colliculus	8	20	1	88	0.008
glomerular layer	1	29	7	38	0.008
granular cell layer	0	18	2	11	0.009
reticular area midbrain	1	6	0	10	0.01
3rd cerebellar lobule	0	0	0	17	0.011
CA1 dorsal hippocampus	0	0	0	24	0.013
periaqueductal gray thalamus	1	2	0	9	0.015
ventral subiculum	0	5	0	8	0.016
root of trigeminal nerve	0	20	3	11	0.017
lateral posterior thalamus	0	0	0	21	0.018
neural lobe pituitary	0	5	0	3	0.018
medial pretectal area	0	0	0	2	0.02
6th cerebellar lobule	0	3	0	51	0.022
ectorhinal ctx	0	0	0	5	0.023
solitary tract nucleus	0	4	0	5	0.023
parafascicular thalamus	0	0	0	1	0.025
lateral amygdala	0	0	0	1	0.031
cochlear area	0	1	0	1	0.033
anterior cingulate ctx	0	7	0	13	0.034
premammillary area	0	3	0	1	0.035
2nd cerebellar lobule	0	0	0	12	0.036
crus 1 of ansiform lobule	0	6	0	61	0.039
principal sensory n. trigeminal	0	24	4	12	0.044
CA1 ventral hippocampus	0	0	0	1	0.044
anterior olfactory nucleus	0	9	0	0	0.046
retrosplenial rostral ctx	0	10	0	107	0.048
lateral orbital ctx	2	0	0	0	0.049
dentate gyrus ventral	0	5	0	4	0.049
primary somatosensory ctx jaw	1	0	0	0	0.051
retrosplenial caudal ctx	0	0	0	13	0.052
CA3 dorsal hippocampus	0	0	0	3	0.057
lemniscal area	0	7	0	2	0.058
medial geniculate	0	10	0	4	0.06
substantia nigra reticularis	0	6	0	7	0.061
habenula area	1	0	0	18	0.061
interpeduncular area	0	0	0	1	0.065
entorhinal ctx	5	19	4	64	0.065
anterior lobe pituitary	5	33	1	34	0.068

Negative BOLD

Region of Interest(ROI)	VEH Med	0.1 Med	0.5 Med	2.5 Med	P val
glomerular layer	0	1	36	32	0.006
external plexiform layer	0	3	14	17	0.007
dorsal medial striatum	0	6	16	6	0.011
granular cell layer	0	0	36	5	0.011
anterior olfactory nucleus	0	0	29	15	0.012
perirhinal ctx	0	3	7	26	0.012
globus pallidus	0	0	0	1	0.019
CA1 dorsal hippocampus	0	14	5	0	0.021
insular ctx	0	4	34	52	0.025
medial geniculate	0	0	6	0	0.027
dentate gyrus ventral	0	0	4	1	0.029
tenia tecta ctx	0	0	3	6	0.036
ventral lateral striatum	0	0	4	7	0.043
ventral medial hypothalamus	0	0	0	3	0.046
ventral pallidum	0	0	9	1	0.047
secondary somatosensory ctx	0	1	1	24	0.048
primary somatosensory ctx upper lip	0	7	11	7	0.051
primary somatosensory ctx forelimb	0	2	6	0	0.054
accumbens shell	0	0	3	0	0.059
olfactory tubercles	0	0	12	11	0.064
zona incerta	0	0	4	0	0.067
lateral hypothalamus	0	0	22	7	0.07
magnocellular preoptic area	0	0	1	0	0.073
anterior cingulate area	0	1	8	11	0.08
prelimbic ctx	0	0	2	0	0.086
substantia innominata	0	0	0	0	0.088
CA3 ventral hippocampus	0	0	6	3	0.091
infralimbic ctx	0	0	7	0	0.092
ventral medial striatum	0	0	0	1	0.096
diagonal band of Broca	0	0	0	1	0.097
CA3 dorsal hippocampus	0	3	6	1	0.1
substantia nigra reticularis	0	0	10	10	0.1
crus 1 of ansiform lobule	0	38	15	25	0.102
primary motor ctx	5	51	24	9	0.102
endopiriform area	0	0	0	0	0.103
dorsal raphe	0	0	0	0	0.109
medial orbital ctx	0	0	4	0	0.113
dentate gyrus dorsal	0	1	6	0	0.114
lateral septum	0	1	21	1	0.114
reticular area midbrain	0	0	3	9	0.117
arcuate area hypothalamus	0	0	0	1	0.12
7th cerebellar lobule	0	0	0	0	0.122
cortical amygdala	0	0	1	1	0.125
primary somatosensory ctx barrel field	0	8	12	10	0.126
trapezoid body	0	0	0	1	0.127
5th cerebellar lobule	1	28	15	0	0.131

medial dorsal thalamus	0	0	0	1	0.076	parvicellular reticular area	0	0	11	13	0.133
imary somatosensory ctx barrel fie	3	0	0	4	0.084	frontal association ctx	0	9	14	5	0.134
posterior thalamus	0	0	0	0	0.088	copula of the pyramis	0	0	3	0	0.14
ventral orbital ctx	0	0	0	0	0.101	periaqueductal gray thalamus	0	7	8	9	0.145
parabrachial area	0	0	0	0	0.112	basal amygdala	0	0	1	0	0.145
10th cerebellar lobule	0	0	0	0	0.115	anterior lobe pituitary	0	0	4	3	0.155
CA3 ventral hippocampus	0	0	0	5	0.116	suprachiasmatic area	0	0	0	0	0.158
copula of the pyramis	0	13	0	4	0.118	CA1 ventral hippocampus	0	0	1	2	0.159
trapezoid body	0	0	0	0	0.12	anterior amygdala	0	0	0	0	0.164
substantia nigra compacta	0	0	0	0	0.12	rostral piriform ctx	0	11	30	28	0.169
intercalated amygdala	0	0	0	0	0.122	visual 1 ctx	0	2	7	0	0.17
anterior hypothalamus	0	0	0	0	0.124	central gray	0	0	9	0	0.171
flocculus cerebellum	1	0	0	1	0.131	primary somatosensory ctx hindlimb	0	0	1	0	0.175
accumbens shell	0	0	0	0	0.132	paramedian lobule	0	0	1	3	0.176
visual 2 ctx	0	2	2	24	0.133	auditory ctx	0	11	13	43	0.183
dentate gyrus dorsal	1	2	0	18	0.133	simple lobule cerebellum	0	10	11	1	0.183
secondary somatosensory ctx	0	0	0	0	0.135	central amygdala	0	0	3	1	0.183
pontine reticular area oral	0	0	0	0	0.136	temporal ctx	1	1	7	8	0.203
sub coeruleus area	0	0	0	0	0.136	pontine reticular area oral	0	0	0	1	0.204
parvicellular reticular area	0	37	6	4	0.136	caudal piriform ctx	0	0	7	1	0.206
9th cerebellar lobule	0	0	0	0	0.139	4th cerebellar lobule	0	4	0	0	0.208
dorsal paragigantocellularis area	0	0	0	0	0.145	lateral geniculate	0	0	0	0	0.21
basal amygdala	0	0	0	1	0.147	interposed area	0	0	0	0	0.215
pontine nuclei	0	7	0	11	0.148	inferior olivary complex	0	0	4	5	0.226
paraflocculus cerebellum	14	29	9	27	0.149	medial amygdala	0	0	7	1	0.243
pedunculopontine tegmental area	0	0	0	0	0.162	ventral orbital ctx	0	0	0	0	0.244
raphe magnus	0	0	0	0	0.162	ventral subiculum	0	0	7	1	0.246
paramedian lobule	0	5	0	9	0.166	lateral amygdala	0	0	0	0	0.246
visual 1 ctx	0	0	6	24	0.166	dorsomedial tegmental area	0	0	0	0	0.253
lateral septum	0	0	0	4	0.176	primary somatosensory ctx jaw	0	18	31	7	0.255
central amygdala	0	0	0	0	0.178	dorsal subiculum	0	0	0	0	0.255
periolivary area	0	0	0	3	0.179	primary somatosensory ctx trunk	0	0	0	0	0.256
medial mammillary area	0	7	0	3	0.184	vestibular area	0	0	6	1	0.274
prelimbic ctx	0	0	0	0	0.186	root of trigeminal nerve	0	0	12	9	0.29
central medial thalamus	0	0	0	0	0.186	ventral tegmental area	0	0	0	0	0.29
ventral pallidum	0	0	0	0	0.187	parietal ctx	0	0	1	0	0.293
auditory ctx	0	4	0	2	0.188	entorhinal ctx	7	37	48	41	0.296
primary motor ctx	3	11	0	16	0.196	lateral cerebellar area	0	0	0	0	0.309
lateral geniculate	0	0	0	4	0.208	paraflocculus cerebellum	4	7	32	13	0.31
insular ctx	8	23	0	4	0.216	gigantocellular reticular area pons	0	0	1	9	0.319
rostral piriform ctx	6	17	28	10	0.221	anterior hypothalamus	0	0	0	0	0.319
caudal piriform ctx	0	1	0	0	0.224	locus ceruleus	0	0	0	0	0.326
supramammillary area	0	0	0	1	0.231	extended amydala	0	0	0	0	0.327
parietal ctx	0	0	0	1	0.234	ventromedial thalamus	0	0	0	0	0.336
ventral tegmental area	0	0	0	0	0.236	visual 2 ctx	0	2	5	4	0.339
magnocellular preoptic area	0	0	0	0	0.244	substantia nigra compacta	0	0	0	0	0.352
anterior amygdala	0	0	0	0	0.25	crus 2 of ansiform lobule	0	0	4	2	0.356
locus ceruleus	0	0	0	0	0.25	red nucleus area	0	0	0	0	0.361
prerubral field	0	0	0	0	0.25	8th cerebellar lobule	0	0	0	0	0.365
subthalamic area	0	0	0	0	0.25	habenula	0	0	0	0	0.374
ventral lateral striatum	0	0	0	1	0.261	principal sensory area trigeminal	0	0	2	5	0.378
medial cerebellar area fastigial	0	0	0	0	0.262	medial cerebellar area fastigial	0	0	0	0	0.379
ventrolateral thalamus	0	0	0	0	0.27	cochlear area	0	4	5	1	0.383

ventral posteriolateral thalamus	0	0	0	0	0.27	posterior hypothalamus	0	0	0	0	0.391
CA2 hippocampus	0	0	0	0	0.27	CA2 hippocampus	0	0	0	0	0.409
tenia tecta ctx	0	10	0	5	0.271	paraventricular hypothalamus	0	0	0	0	0.427
olfactory tubercles	0	2	0	0	0.276	prerubral field	0	0	0	0	0.427
8th cerebellar lobule	0	0	0	5	0.287	periolivary area	0	0	0	3	0.435
dorsal raphe	0	0	0	0	0.292	median raphe	0	0	0	0	0.438
central gray	0	0	0	0	0.295	claustrum	0	0	0	0	0.441
medial orbital ctx	0	1	0	1	0.299	lemniscal area	0	0	0	2	0.441
anterior thalamus	0	0	0	1	0.301	lateral posterior thalamus	0	0	0	0	0.449
inferior olivary complex	0	0	0	0	0.305	ventrolateral thalamus	0	0	0	0	0.464
secondary motor ctx	0	16	0	12	0.307	medial septum	0	0	0	0	0.465
raphe linear	0	0	0	0	0.308	dorsal medial hypothalamus	0	0	0	0	0.475
perirhinal ctx	0	6	0	0	0.31	premammillary area	0	0	0	0	0.481
ventral medial hypothalamus	0	2	0	0	0.312	secondary motor ctx	4	39	19	21	0.485
dorsomedial tegmental area	0	0	0	0	0.324	parabrachial area	0	0	0	0	0.487
accumbens core	0	0	0	0	0.33	subthalamic area	0	0	0	0	0.488
medial amygdala	0	4	0	3	0.34	medial preoptic area	0	0	0	0	0.49
lateral dorsal thalamus	0	0	0	0	0.346	inferior colliculus	1	10	20	2	0.492
interposed area	0	0	0	0	0.352	lateral orbital ctx	0	0	7	2	0.494
endopiriform area	0	0	0	0	0.355	parafascicular thalamus	0	0	0	0	0.503
reuniens area	0	0	0	0	0.355	retrosplenial rostral ctx	2	8	15	10	0.508
ventral medial striatum	0	0	0	0	0.361	dorsal lateral striatum	0	0	0	1	0.512
ventral anterior thalamus	0	0	0	0	0.366	supraoptic hypothalamus	0	0	0	0	0.514
paraventricular hypothalamus	0	0	0	0	0.374	10th cerebellar lobule	0	0	0	0	0.515
paraventricular thalamus	0	0	0	0	0.379	ventral posteriomedial thalamus	0	0	0	0	0.52
crus 2 of ansiform lobule	0	0	0	1	0.384	raphe obscurus area	0	0	0	0	0.521
retrochiasmatic area	0	0	0	0	0.39	1st cerebellar lobule	0	0	0	0	0.523
pontine reticular area caudal	0	0	0	0	0.405	pineal gland	0	0	0	0	0.536
suprachiasmatic area	0	0	0	0	0.418	raphe magnus	0	0	0	0	0.537
7th cerebellar lobule	0	0	0	0	0.419	lateral preoptic area	0	0	0	0	0.541
triangular septal area	0	0	0	0	0.424	paraventricular thalamus	0	0	0	0	0.553
frontal association ctx	3	3	0	1	0.437	superior colliculus	0	12	5	1	0.562
medial preoptic area	0	0	0	0	0.447	triangular septal area	0	0	0	0	0.563
lateral hypothalamus	0	15	3	11	0.459	precuniform area	0	0	0	0	0.566
cortical amygdala	0	2	1	0	0.47	reticulotegmental area	0	0	0	0	0.575
dorsal medial hypothalamus	0	0	0	0	0.47	pontine nuclei	0	0	0	2	0.58
claustrum	0	0	0	0	0.475	3rd cerebellar lobule	0	2	4	1	0.586
median raphe	0	0	0	0	0.475	facial nucleus area	0	0	0	1	0.599
precuniform area	0	0	0	0	0.475	reuniens area	0	0	0	0	0.611
reticulotegmental area	0	0	0	0	0.475	reticular thalamus	0	1	0	0	0.611
ventromedial thalamus	0	0	0	0	0.475	bed nucleus stria terminalis	0	0	0	0	0.622
supraoptic hypothalamus	0	0	0	0	0.498	dorsal paragigantocellularis area	0	0	0	0	0.626
pineal gland	0	0	0	1	0.504	raphe linear	0	0	0	0	0.635
lateral cerebellar area	0	0	0	0	0.527	posterior thalamus	0	0	0	0	0.636
rimary somatosensory ctx hindlim	0	0	0	0	0.532	neural lobe pituitary	0	0	0	0	0.647
temporal ctx	0	0	0	1	0.533	2nd cerebellar lobule	0	1	1	0	0.654
1st cerebellar lobule	0	0	0	0	0.554	ventral posteriolateral thalamus	0	0	0	0	0.654
facial area	0	0	0	0	0.558	accumbens core	0	0	0	0	0.657
rimary somatosensory ctx should	0	0	0	0	0.57	intercalated amygdala	0	0	0	0	0.671
gigantocellular reticular area pons	0	4	0	3	0.573	medial pretectal area	0	0	0	0	0.685
diagonal band of Broca	0	0	0	0	0.583	supramammillary area	0	0	0	0	0.691
primary somatosensory ctx trunk	0	0	0	0	0.586	solitary tract nucleus	0	0	0	0	0.692
zona incerta	0	0	0	0	0.59	retrochiasmatic area	0	0	0	0	0.705
red area	0	0	0	0	0.599	sub coeruleus area	0	0	0	0	0.736
globus pallidus	0	0	0	0	0.626	interpeduncular area	0	0	0	0	0.74
medial septum	0	0	0	0	0.644	ventral anterior thalamus	0	0	0	0	0.745
rimary somatosensory ctx forelim	0	0	0	0	0.656	6th cerebellar lobule	0	0	0	0	0.753
dorsal subiculum	1	1	0	5	0.679	central medial thalamus	0	0	0	0	0.754
rimary somatosensory ctx upper li	0	0	0	0	0.687	medial mammillary area	0	0	0	0	0.77

infralimbic ctx	0	0	0	0	0.695	9th cerebellar lobule	0	0	0	0	0.771
reticular area	0	0	0	0	0.695	flocculus cerebellum	0	2	0	0	0.785
dorsal medial striatum	0	0	0	0	0.697	anterior thalamus	0	0	0	0	0.814
ventral posteriomedial thalamus	0	0	0	0	0.729	medial dorsal thalamus	0	0	0	0	0.816
motor trigeminal area	0	0	0	0	0.733	pedunculopontine tegmental area	0	0	0	0	0.817
substantia innominata	0	0	0	0	0.749	anterior pretectal area	0	0	0	0	0.843
extended amydala	0	0	0	0	0.76	retrosplenial caudal ctx	0	4	0	3	0.899
bed nucleus stria terminalis	0	0	0	0	0.781	pontine reticular n. caudal	0	0	0	0	0.907
dorsal lateral striatum	0	0	0	0	0.821	ectorhinal ctx	1	0	0	0	0.946
posterior hypothalamic area	0	0	0	0	0.933	primary somatosensory ctx shoulder	0	0	0	0	0.97
raphe obscurus area	0	0	0	0	0.988	motor trigeminal area	0	0	0	0	0.987

Table 3S | Brain Activation with Intraperitoneal Oxytocin 10 Minutes Post Injection.
Shown are 171 brain areas and their median (med) number of positive and negative voxels affected 10 min following IP injections of vehicle (Veh n = 12), 0.1 (n = 9), 0.5 (n = 13) and 2.5 (n = 12) mg OT. The regions of interest are rank order for significance. Probability values are presented on the far right column. The red and blue highlight the significantly activated areas for positive and negative BOLD, respectively. The voxel numbers for all four conditions were analyzed using a Newman-Keuls multiple comparisons test statistic. The yellow highlights mark brain areas that comprise the OT receptor system shown in Fig 1.
