# Supplementary material for: Distinct BOLD Activation Profiles Following Central and Peripheral Oxytocin Administration in Awake Rats
Source: Front Behav Neurosci. 2015 Sep 17;9:245. doi: 10.3389/fnbeh.2015.00245 (PMC4585275; doi:10.3389/fnbeh.2015.00245)
Supplement: Supplementary file 4 [file Table_4.PDF]

TABLE 4S **Volume of Activation 20 Min Post IP Oxytocin**

Positive BOLD

Region of Interest(ROI)	VEH Med	0.1 Med	0.5 Med	2.5 Med	P val
external plexiform layer	1	19	17	26	0.002
dorsal raphe	0	0	0	2	0.009
glomerular layer	10	63	51	79	0.01
cortical amygdala	2	15	13	4	0.011
perirhinal ctx	3	16	16	4	0.012
lateral orbital ctx	11	6	0	0	0.013
accumbens shell	0	6	1	3	0.015
rostral piriform ctx	30	71	70	22	0.016
posterior thalamus	0	4	0	0	0.02
ectorhinal ctx	0	0	1	0	0.034
ventromedial thalamus	0	1	0	0	0.053
reticular area midbrain	11	33	14	18	0.055
4th cerebellar lobule	0	1	9	43	0.08
solitary tract nucleus	0	5	2	9	0.081
CA1 ventral hippocampus	0	6	2	2	0.086
accumbens core	0	0	0	0	0.088
granular cell layer	7	25	25	34	0.09
8th cerebellar lobule	0	0	0	13	0.094
median raphe	0	0	0	0	0.094
olfactory tubercles	1	53	15	23	0.096
ventral posteriomedial thalamus	0	0	0	0	0.099
parafascicular thalamus	0	4	1	2	0.104
ventral pallidum	0	1	0	2	0.11
lateral preoptic area	0	1	0	1	0.11
5th cerebellar lobule	16	17	32	73	0.114
motor trigeminal area	0	0	0	0	0.115
periaqueductal gray thalamus	5	15	5	23	0.121
parabrachial area	0	2	1	4	0.121
bed nucleus stria terminalis	1	6	0	2	0.122
entorhinal ctx	43	66	93	73	0.123
medial geniculate	2	19	8	9	0.124
anterior hypothalamic area	0	5	0	0	0.127
zona incerta	0	7	1	1	0.128
superior colliculus	50	69	59	82	0.134
medial pretectal area	0	0	0	0	0.136
anterior olfactory nucleus	12	14	4	10	0.14
root of trigeminal nerve	10	33	19	21	0.141
substantia nigra compacta	0	1	0	1	0.149
lateral dorsal thalamus	2	0	0	0	0.151
endopiriform area	0	0	0	0	0.154
medial amygdala	1	16	15	16	0.167
triangular septal area	0	0	0	2	0.174
vestibular area	3	13	16	22	0.175
principal sensory area trigeminal	2	18	22	27	0.179
CA2 hippocampus	0	0	0	0	0.179
arcuate hypothalamus	0	4	3	4	0.179

Negative BOLD

Region of Interest(ROI)	VEH Med	0.1 Med	0.5 Med	2.5 Med	P val
external plexiform layer	0	27	25	32	0.004
secondary somatosensory ctx	0	6	26	52	0.006
auditory ctx	3	37	106	114	0.022
dorsal medial striatum	0	40	28	13	0.024
perirhinal ctx	12	13	12	50	0.027
olfactory tubercles	0	0	27	21	0.027
CA3 dorsal hippocampus	0	28	20	7	0.028
glomerular layer	1	32	35	39	0.033
insular ctx	4	14	84	104	0.041
primary somatosensory ctx upper lip	0	12	28	35	0.051
diagonal band of Broca	0	0	0	1	0.051
rostral piriform ctx	5	23	85	78	0.056
granular cell layer	0	51	53	36	0.066
substantia nigra reticularis	1	0	5	11	0.068
ectorhinal ctx	8	12	3	14	0.077
3rd cerebellar lobule	7	18	0	6	0.08
neural lobe pituitary	0	0	0	0	0.087
1st cerebellar lobule	0	3	0	0	0.089
central gray	0	8	6	0	0.1
dorsal raphe	0	0	1	0	0.105
cortical amygdala	0	0	0	6	0.127
tenia tecta ctx	0	0	6	0	0.127
4th cerebellar lobule	6	20	0	5	0.129
caudal piriform ctx	0	6	20	13	0.142
ventral subiculum	0	4	7	8	0.147
lateral hypothalamus	0	2	18	35	0.15
periaqueductal gray thalamus	2	18	34	7	0.157
suprachiasmatic area	0	0	0	0	0.158
anterior lobe pituitary	0	0	0	4	0.17
trapezoid body	0	0	0	2	0.17
gigantocellular reticular area pons	12	5	5	29	0.178
red nucleus area	0	0	0	1	0.18
paraflocculus cerebellum	13	44	26	45	0.182
substantia nigra compacta	0	0	0	2	0.187
primary somatosensory ctx barrel field	0	43	60	26	0.187
primary somatosensory ctx forelimb	1	22	7	10	0.188
raphe linear	0	0	0	0	0.189
CA3 ventral hippocampus	0	2	5	4	0.191
basal amygdala	0	0	6	7	0.193
zona incerta	0	2	2	7	0.199
substantia innominata	0	0	0	0	0.202
CA1 ventral hippocampus	1	0	2	10	0.205
entorhinal ctx	69	115	76	130	0.206
supraoptic area hypothalamus	0	0	0	0	0.207
anterior olfactory nucleus	1	30	55	29	0.211
parvicellular reticular area	15	3	18	32	0.213

caudal piriform ctx	0	15	3	2	0.181
ventral subiculum	9	27	15	17	0.191
extended amydala	0	0	0	0	0.202
anterior pretectal area	1	5	5	7	0.207
dorsal paragigantocellularis area	0	0	1	2	0.209
CA1 dorsal hippocampus	14	6	21	24	0.209
magnocellular preoptic area	0	1	0	1	0.212
3rd cerebellar lobule	4	5	9	17	0.218
intercalated amygdala	0	0	0	0	0.218
crus 1 of ansiform lobule	10	34	45	88	0.224
ventral medial striatum	0	12	0	4	0.225
dorsomedial tegmental area	0	0	0	1	0.228
simple lobule cerebellum	13	11	28	35	0.233
primary somatosensory ctx jaw	12	0	0	0	0.239
10th cerebellar lobule	0	0	5	3	0.241
reuniens area	0	0	0	0	0.25
trapezoid body	0	1	0	0	0.254
dorsal medial hypothalamus	0	0	0	0	0.258
raphe linear	0	0	0	0	0.269
primary somatosensory ctx trunk	0	0	0	0	0.27
pontine reticular area oral	0	0	0	0	0.282
interpeduncular area	0	7	0	5	0.288
anterior thalamus	6	3	0	2	0.288
premammillary area	1	4	1	2	0.294
copula of the pyramis	6	20	8	18	0.296
medial mammillary area	2	13	5	9	0.297
pontine nuclei	1	27	6	22	0.309
auditory ctx	4	8	7	3	0.318
lateral amygdala	0	1	0	0	0.324
pedunculopontine tegmental area	0	0	0	0	0.331
dentate gyrus ventral	4	27	11	18	0.333
2nd cerebellar lobule	8	13	8	26	0.338
7th cerebellar lobule	0	0	0	8	0.341
basal amygdala	0	4	4	1	0.345
lateral septum	19	12	6	26	0.35
claustrum	0	0	0	0	0.353
central amygdala	0	4	1	3	0.353
red nucleus area	0	0	0	0	0.358
lateral hypothalamus	5	52	27	28	0.365
ventral lateral striatum	0	4	1	3	0.384
raphe obscurus area	0	0	0	0	0.397
lateral geniculate	2	10	3	4	0.401
cochlear area	0	0	4	3	0.403
diagonal band of Broca	0	4	0	5	0.41
ventrolateral thalamus	0	0	0	0	0.41
ventral medial hypothalamus	1	6	0	3	0.42
lemniscal area	3	11	7	10	0.432
paramedian lobule	5	11	3	24	0.433
reticular thalamus	1	2	0	2	0.444
ventral tegmental area	0	2	0	3	0.448
inferior colliculus	33	44	72	93	0.458
subthalamic area	0	0	0	0	0.461
ventral anterior thalamus	0	0	0	0	0.461

reticular area midbrain	2	7	13	18	0.219
primary motor ctx	28	95	75	54	0.22
paraventricular thalamus	0	0	0	2	0.232
primary somatosensory ctx jaw	2	62	59	13	0.232
paraventricular hypothalamus	0	0	0	0	0.232
reticulotegmental area	0	0	0	0	0.236
primary somatosensory ctx hindlimb	0	11	3	10	0.24
periolivary area	2	0	6	4	0.248
reticular area midbrain	0	10	4	2	0.251
ventral pallidum	0	0	6	8	0.261
lateral dorsal thalamus	0	1	0	0	0.265
simple lobule cerebellum	12	69	7	32	0.283
anterior thalamus	0	1	0	12	0.287
lateral septum	2	24	18	15	0.287
visual 2 ctx	1	96	42	27	0.288
parietal ctx	0	20	2	2	0.29
5th cerebellar lobule	42	65	3	24	0.293
extended amydala	0	0	0	1	0.295
accumbens shell	0	0	2	3	0.309
dorsal lateral striatum	1	41	12	6	0.318
CA1 dorsal hippocampus	6	36	25	9	0.323
ventral tegmental area	0	0	0	1	0.326
anterior cingulate ctx	15	27	24	30	0.334
anterior hypothalamus	0	0	0	0	0.344
lateral orbital ctx	1	10	15	20	0.348
globus pallidus	0	1	0	2	0.35
lateral geniculate	0	2	7	0	0.356
visual 1 ctx	3	51	25	20	0.36
2nd cerebellar lobule	2	5	1	4	0.37
retrosplenial caudal ctx	3	19	12	20	0.37
ventral lateral striatum	1	25	15	49	0.373
7th cerebellar lobule	0	0	0	0	0.374
crus 1 of ansiform lobule	17	52	30	83	0.389
inferior olivary complex	0	0	0	4	0.391
posterior hypothalamus	0	0	0	1	0.393
pineal gland	0	0	0	0	0.395
anterior amygdala	0	0	0	0	0.396
inferior colliculus	22	72	15	51	0.399
habenula	0	0	0	1	0.399
dentate gyrus dorsal	0	12	6	5	0.41
facial nucleus area	0	6	0	7	0.411
endopiriform area	0	2	7	10	0.412
dorsal subiculum	0	4	5	0	0.425
magnocellular preoptic area	0	0	0	0	0.425
retrochiasmatic area	0	0	0	0	0.43
accumbens core	0	0	0	2	0.434
triangular septal area	0	2	0	0	0.436
ventral posteriomedial thalamus	0	0	0	3	0.437
intercalated amygdala	0	0	0	0	0.439
6th cerebellar lobule	2	36	4	46	0.44
primary somatosensory ctx trunk	0	1	1	0	0.448
dentate gyrus ventral	1	0	2	5	0.456
claustrum	0	0	0	3	0.463

anterior amygdala	0	0	0	0	0.468
locus ceruleus	0	0	0	0	0.47
medial preoptic area	0	3	0	7	0.472
ventral posteriolateral thalamus	0	0	0	0	0.474
neural lobe pituitary	3	7	5	7	0.478
anterior lobe pituitary	27	51	45	57	0.48
medial septum	0	0	0	0	0.483
supramammillary area	0	0	0	2	0.484
6th cerebellar lobule	6	20	32	45	0.491
medial dorsal thalamus	3	5	1	2	0.5
crus 2 of ansiform lobule	8	5	0	10	0.508
lateral posterior thalamus	4	14	16	22	0.524
reticulotegmental area	0	0	0	0	0.527
central medial thalamus	0	0	0	0	0.529
retrosplenial rostral ctx	37	32	81	73	0.532
primary somatosensory ctx shoulder	0	0	0	0	0.542
visual 1 ctx	7	7	17	32	0.544
primary somatosensory ctx forelimb	0	0	0	0	0.548
dorsal lateral striatum	0	2	0	5	0.554
retrochiasmatic area	0	1	0	1	0.561
posterior hypothalamus	0	1	1	0	0.579
inferior olivary complex	3	2	6	0	0.584
gigantocellular reticular area pons	1	11	32	9	0.585
9th cerebellar lobule	0	1	0	2	0.589
globus pallidus	0	0	0	0	0.589
periolivary area	0	2	0	3	0.593
temporal ctx	0	0	0	0	0.595
pineal gland	0	0	1	1	0.599
supraoptic area hypothalamus	0	0	0	0	0.606
lateral cerebellar area	0	0	0	0	0.608
prelimbic ctx	3	18	0	3	0.61
visual 2 ctx	0	6	3	10	0.617
ventral orbital ctx	0	0	0	0	0.63
suprachiasmatic area	0	0	0	0	0.632
facial nucleus area	0	1	5	2	0.639
parvicellular reticular area	4	31	14	35	0.64
insular ctx	26	18	21	14	0.642
primary motor ctx	8	15	0	9	0.654
dorsal medial striatum	0	0	1	5	0.666
paraflocculus cerebellum	41	64	36	45	0.667
retrosplenial caudal ctx	0	0	5	10	0.668
parietal ctx	0	0	0	0	0.68
primary somatosensory ctx hindlimb	0	0	0	0	0.687
paraventricular thalamus	4	7	2	4	0.691
flocculus cerebellum	2	2	0	2	0.702
infralimbic ctx	8	14	4	10	0.755
habenula	10	12	8	22	0.767
secondary somatosensory ctx	3	1	3	0	0.771
tenia tecta ctx	27	29	14	28	0.782
substantia nigra reticularis	9	22	7	18	0.785
pontine reticular area caudal	0	2	0	0	0.796
frontal association ctx	9	10	4	9	0.796
central gray	3	0	0	2	0.839
anterior cingulate ctx	0	11	12	13	0.84
raphe magnus	0	0	0	0	0.841
primary somatosensory ctx barrel field	3	4	0	1	0.853
paraventricular hypothalamus	0	0	0	0	0.873
substantia innominata	0	0	0	0	0.874
secondary motor ctx	4	21	7	9	0.874

precuniform area	0	0	0	0	0.467
ventral medial hypothalamus	0	0	1	2	0.473
dorsal paragigantocellularis area	0	3	0	0	0.474
dorsal medial hypothalamus	0	0	0	0	0.475
reuniens area	0	0	0	0	0.477
medial amygdala	1	1	1	3	0.482
lateral posterior thalamus	0	2	3	0	0.484
raphe magnus	0	0	0	1	0.5
medial orbital ctx	0	0	1	0	0.511
prelimbic ctx	0	0	5	4	0.513
frontal association ctx	3	18	24	27	0.515
raphe obscurus area	0	0	0	0	0.516
secondary motor ctx	41	78	38	83	0.522
interpeduncular area	0	0	0	0	0.53
dorsomedial tegmental area	0	0	3	0	0.531
flocculus cerebellum	1	3	0	4	0.537
temporal ctx	16	11	22	27	0.554
ventral orbital ctx	0	3	2	1	0.554
medial geniculate	0	4	8	4	0.558
medial septum	0	0	0	0	0.565
pontine reticular area caudal	4	2	2	8	0.566
root of trigeminal nerve	8	12	23	24	0.567
primary somatosensory ctx shoulder	0	2	0	0	0.567
8th cerebellar lobule	0	0	0	0	0.569
infralimbic ctx	0	0	1	1	0.58
pontine nuclei	3	0	4	4	0.582
bed nucleus stria terminalis	0	0	0	0	0.585
interposed area	0	0	0	0	0.588
pontine reticular area oral	1	0	0	2	0.606
motor trigeminal area	0	0	0	0	0.608
lemniscal area	1	2	3	8	0.609
lateral cerebellar area	0	0	0	0	0.611
central medial thalamus	0	0	0	0	0.621
vestibular area	4	17	3	2	0.626
crus 2 of ansiform lobule	0	0	1	10	0.63
retrosplenial rostral ctx	15	95	54	65	0.632
supramammillary area	0	0	0	0	0.643
9th cerebellar lobule	0	0	0	0	0.644
ventromedial thalamus	0	0	0	2	0.647
lateral amygdala	0	1	5	1	0.657
central amygdala	5	6	9	9	0.659
prerubral field	0	0	0	0	0.663
cochlear area	0	6	0	5	0.674
principal sensory area trigeminal	11	7	19	18	0.692
ventral medial striatum	0	1	0	2	0.7
ventral posteriolateral thalamus	0	2	0	2	0.702
parabrachial area	0	0	2	0	0.717
arcuate area hypothalamus	0	0	0	0	0.722
paramedian lobule	0	4	5	9	0.734
pedunculopontine tegmental area	0	0	0	0	0.744
medial dorsal thalamus	0	0	0	1	0.748
10th cerebellar lobule	1	0	0	0	0.752
ventral anterior thalamus	0	0	0	0	0.77
posterior thalamus	0	2	0	3	0.773
solitary tract area	0	0	0	2	0.775
lateral preoptic area	0	0	0	0	0.785
CA2 hippocampus	0	0	0	1	0.792
sub coeruleus area	0	0	1	1	0.796
ventrolateral thalamus	0	3	3	0	0.8

**sub coeruleus area**	**0**	**0**	**0**	**0**	**0.901**	**medial preoptic area**	**0**	**0**	**0**	**0**	**0.823**
**CA3 ventral hippocampus**	**7**	**17**	**13**	**14**	**0.913**	**medial mammillary area**	**0**	**0**	**0**	**0**	**0.84**
**primary somatosensory ctx upper lip**	**5**	**3**	**1**	**8**	**0.921**	**superior colliculus**	**16**	**41**	**23**	**28**	**0.87**
**medial cerebellar area fastigial**	**0**	**0**	**0**	**0**	**0.923**	**parafascicular thalamus**	**0**	**0**	**0**	**0**	**0.876**
**dentate gyrus dorsal**	**20**	**22**	**11**	**33**	**0.933**	**premammillary area**	**0**	**0**	**0**	**0**	**0.897**
**CA3 dorsal hippocampus**	**5**	**5**	**2**	**8**	**0.947**	**copula of the pyramis**	**1**	**0**	**1**	**4**	**0.908**
**interposed area**	**0**	**0**	**0**	**0**	**0.971**	**medial cerebellar area fastigial**	**0**	**0**	**0**	**0**	**0.965**
**1st cerebellar lobule**	**0**	**0**	**0**	**0**	**0.979**	**subthalamic area**	**0**	**0**	**0**	**0**	**0.979**
**medial orbital ctx**	**5**	**13**	**6**	**4**	**0.993**	**anterior pretectal area**	**0**	**0**	**0**	**0**	**0.986**
**dorsal subiculum**	**18**	**17**	**11**	**12**	**1**	**locus ceruleus**	**0**	**0**	**0**	**0**	**0.988**

Table 4S | Brain Activation with Intraperitoneal Oxytocin 20 Minutes Post Injection.
Shown are 171 brain areas and their median (med) number of positive and negative voxels affected 10 min following IP injections of vehicle (Veh n = 12), 0.1 (n = 9), 0.5 (n = 13) and 2.5 (n = 12) mg OT. The regions of interest are rank order for significance. Probability values are presented on the far right column. The red and blue highlight the significantly activated areas for positive and negative BOLD, respectively. The voxel numbers for all four conditions were analyzed using a Newman-Keuls multiple comparisons test statistic. The yellow highlights mark brain areas that comprise the OT receptor system shown in Fig 1.
